# Spatiotemporal dynamics of self-assembled structures in enzymatically induced agonistic and antagonistic conditions†

**DOI:** 10.1039/d1sc05353a

**Published:** 2021-11-22

**Authors:** Priyanka, Ekta Shandilya, Surinder Kaur Brar, Rishi Ram Mahato, Subhabrata Maiti

**Affiliations:** Department of Chemical Sciences, Indian Institute of Science Education and Research (IISER) Mohali Knowledge City Manauli 140306 India smaiti@iisermohali.ac.in

## Abstract

Predicting and designing systems with dynamic self-assembly properties in a spatiotemporal fashion is an important research area across disciplines ranging from understanding the fundamental non-equilibrium features of life to the fabrication of next-generation materials with life-like properties. Herein, we demonstrate a spatiotemporal dynamics pattern in the self-assembly behavior of a surfactant from an unorganized assembly, induced by adenosine triphosphate (ATP) and enzymes responsible for the degradation or conversion of ATP. We report the different behavior of two enzymes, alkaline phosphatase (ALP) and hexokinase (HK), towards adenosine triphosphate (ATP)-driven surfactant assembly, which also results in contrasting spatiotemporal dynamic assembly behavior. Here, ALP acts antagonistically, resulting in transient self-assemblies, whereas HK shows agonistic action with the ability to sustain the assemblies. This dynamic assembly behavior was then used to program the time-dependent emergence of a self-assembled structure in a two-dimensional space by maintaining concentration gradients of the enzymes and surfactant at different locations, demonstrating a new route for obtaining ‘spatial’ organizational adaptability in a self-organized system of interacting components for the incorporation of programmed functionality.

## Abstract 2

We have shown ATP-driven spatiotemporally distinct self-organization pattern of a surfactant in a two-dimensional space using enzymes, demonstrating a new route for obtaining ‘spatial’ organizational adaptability among interacting components.

## Graphical abstract

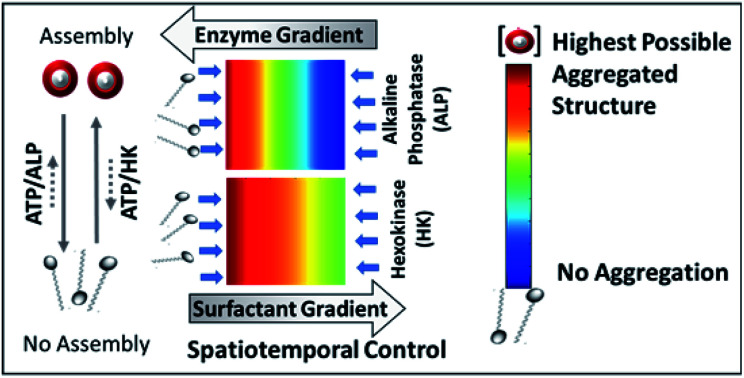

## Introduction

Spatiotemporally dynamic assemblies generated through a synergistic paradigm among interactive chemical components can often result in the emergence of complicated and unpredictable phenomena.^1,2^ This area has been advanced as one of the most important scientific problems for understanding complex networking patterns in nature, such as connectivity in living cells or neuronal networks or the creation of next-generation materials with perceptual stability.^3–9^ The agonistic or antagonistic relationships among the different constituents are the key factors or switches between the up- and downregulation of the overall dynamic processes.^10–14^ In many cases, reaction–diffusion related to oscillatory systems has been used to create non-equilibrium assemblies and thereby surface patterns.^11–14^ In the last decade or so, chemists have also successfully developed dynamic self-organized systems with certain exemplary characteristics, such as self-sorting, self-replication, the ability to utilize both physical and chemical energy from the environment, and motility or taxis, *etc.*^15–22^ In this context, many functional chemical-fuel-driven transiently formed self-assembled systems inspired by the dynamic self-organization of the proteins tubulin or actin (fueled by guanosine triphosphate (GTP) or adenosine triphosphate (ATP)) have been reported.^23–26^ In fact, these systems have allowed access to temporal control over a range of diverse properties, including chemical reactivity, material strength, time-dependent drug delivery, and optoelectronic properties, among others.^27–34^

**Fig. 1:**
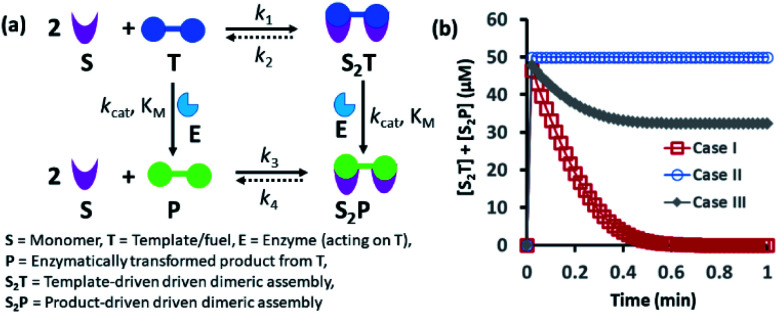
(a) Schematic representation of the dynamic assembly of the dimers S_2_T and S_2_P from the monomer (S), mediated by the fuel (T) and fuel that has been enzymatically transformed into the product (P), when all the pathways are working simultaneously. (b) Kinetic profile was generated through kinetic modelling using Python (details are in Fig. S1 and ESI†) and for the system [S] = 200 μM, [T] = 50 μM with the rate constant values *k*_1_ = 10^2^ μM^−2^ h^−1^, *k*_2_ = 0.01 h^−1^, *k*_cat_ = 250 h^−1^ (*k*_cat_ is the turnover number of the enzyme where [E] is fixed at 1 μM for all the cases) and *K*_M_ = 50 μM for all cases; only the *k*_3_ and *k*_4_ values were changed. Case I: *k*_3_ = 10^−4^ μM^−2^ h^−1^, *k*_4_ = 10^4^ h^−1^; case II: *k*_3_ = 10^4^ μM^−2^ h^−1^, *k*_4_ = 10^−4^ h^−1^; case III: *k*_3_ = 10^−2^ μM^−2^ h^−1^, *k*_4_ = 10^2^ h^−1^.

In many instances, enzymes have also been used for the development of transient synthetic chemical assemblies in response to light, pH, *etc.* In principle, enzymes capable of dissociating the aggregate-stabilizing chemical fuel can generate different types of dynamic instability in the system (Fig. 1).^35–40^ Here, the binding of the chemical fuel and the building blocks is a faster process than the degradation of the chemical fuel by the enzyme, and in general, the products generated by the chemical fuel are not able to stabilize the aggregates of the building block to sustain the organized assembly. Fig. 1 depicts two monomers (S) that are only able to form a dimeric assembly (S_2_T) in presence of the fuel (T), which is a substrate of the enzyme (E). Due to the presence of E, T and S_2_T can both also be converted to the product (P) and S_2_P (dimeric assembly of S mediated by P). S_2_P can then either dissociate to the monomer (S) and P or remain stable in the aggregate form if P also has templating ability. In Fig. 1b, we have illustrated the possible outcomes of these processes by assuming three different cases. For case I, *k*_3_ ≪ *k*_4_, which implies that the dissociation rate of S_2_P is much higher than that of association between S and P; in other words, P does not have any aggregate-stabilizing ability, and thus transient formation of the assembly (here, S_2_T) is observed (Fig. 1b).^36–38^ However, when *k*_3_ is nearly equal to or higher than *k*_4_, P also has aggregate-stabilizing ability to sustain the assembly as a mixture of S_2_T and S_2_P or only S_2_P, as shown in cases II and III (Fig. 1b and S1, ESI†). In this manuscript, we have termed cases II and III as the agonistic condition, as in these cases, even after enzymatic conversion, the assembled structure remained over time. Similarly, the scenario described in case I is referred to as the antagonistic condition, which has also been termed as self-assembly under dissipative conditions in previous reports in the literature.^40^ However, until now, the propagation and sustainability of chemical-fuel-driven organized systems or transiently evolved systems due to a catalytic process have not been explored in detail in a synthetic system, particularly in terms of their spatial organization over time.^41–47^ It is worth mentioning that these kinds of systems are ubiquitous and significant in cellular systems, *e.g.*, the spatiotemporal dynamics of proteomes, metabolites, and the formation and growth of protein assemblies.^48–52^

**Fig. 2:**
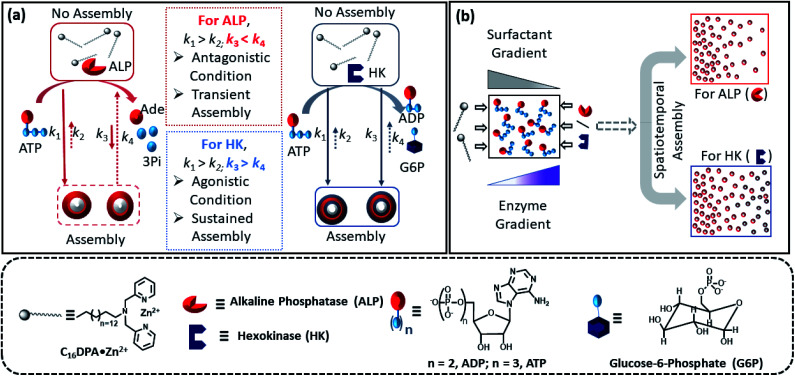
(a) Schematic representation of the different types of ATP-driven C_16_DPA·Zn^2+^ surfactant assembly formation when ALP and HK are used separately. The glucose and Mg(NO_3_)_2_ in the system are not shown in the schematic for simplicity. Solid arrows denote assembly formation, whereas dashed arrows denote dissociation; the extent of association/dissociation is denoted by the length of the arrow. (b) Schematic representation of the spatiotemporal assembly formation of the surfactant templated by ATP when gradients of the surfactant and ALP or HK are maintained from opposite sides.

Herein, we report how the presence of two enzymes – hexokinase (HK) and alkaline phosphatase (ALP) – can create agonistic and antagonistic self-assembly events with a surfactant (C_16_DPA·Zn^2+^, designated as S) containing a dipicolylamine-bound zinc complex (DPA·Zn^2+^) headgroup in the presence of adenosine triphosphate (ATP) under similar experimental conditions (Fig. 2a). HK helps to sustain the self-assembly response of the system, as it converts ATP to adenosine diphosphate (ADP) and glucose-6-phosphate (G6P), thereby creating an agonistic condition for the self-assembly. On the contrary, the ALP-hydrolyzed products adenosine (Ade) and phosphate (Pi) do not have the ability to maintain the self-assembly response; therefore, we termed this as the antagonistic condition. Additionally, we have shown how the generation of population dynamics of the self-assembled units emerged under both agonistic and antagonistic conditions as a function of time and space. In one case, concentration gradients of the surfactant, which is the building block of the assemblies and the major self-assembly regulating factor, and the enzyme were maintained from two different locations in a system with a uniform substrate concentration (Fig. 2b). In the other case, the spatiotemporal aggregation behavior of a uniform distribution of the ATP-stabilizing surfactant in a two-dimensional space was observed while simultaneously generating agonistic and antagonistic conditions by employing concentration gradients of HK and ALP from different sides. We also theoretically investigated the process by assuming a simplistic reaction–diffusion system in a 2-D space based on the equations used in Fig. 1a, where S (the surfactant in monomeric or unassembled form) can aggregate into S_2_T or S_2_P in the presence of the fuel T or P (akin to the formation of surfactant aggregates fuelled by ATP or the possible formation of surfactant aggregates driven by the ALP- or HK-transformed products).

## Results and discussion

First, we synthesized the metallosurfactant (C_16_DPA·Zn^2+^), which contains a dipicolylamine-bound zinc complex (DPA·Zn^2+^) headgroup (Fig. 2, also see ESI† for details). The DPA·Zn^2+^ complex is well known for its selective anion recognition properties (especially toward phosphates) and has also been used for generating various dynamic supramolecular motifs.^53,54^ Initially, we measured the critical aggregation concentration (CAC) of the C_16_DPA·Zn^2+^ surfactant using conductivity and scattering intensity (count rate) *via* dynamic light scattering (DLS) assay at a fixed attenuator, *i.e.*, source laser intensity.^55^ From these experiments, the CAC value of the surfactant was found to be approximately 35 ± 5 μM (Fig. S5 and S6, ESI†). We observed reproducible DLS data with a good count rate only after using 50 μM of the surfactant during our titration (Fig. S7, ESI†).

Based on the above-described results, we chose a C_16_DPA·Zn^2+^ concentration of 25 μM for the rest of our studies, as the surfactant alone does not form assemblies at this concentration. However, the introduction of ATP into the system could potentially result in assembly formation due to its stronger ability to bind as a counter-ion with the Zn^2+^ containing head group; notably, this strategy has been used in previous reports in the literature.^28,56,57^ To introduce dynamicity in the assembly formation, we used the enzymes ALP and HK, which can convert ATP to adenosine (Ade) + three molecules of inorganic phosphate (Pi) or adenosine diphosphate (ADP) + glucose-6-phosphate (G6P), respectively, under our experimental conditions in the presence of 1 mM glucose and 0.5 mM Mg(NO_3_)_2_. Mg^2+^ acts as the co-factor of the HK enzyme and is also known to increase the activity of ALP. Both these enzymatic conversions were confirmed using HPLC under our experimental conditions (Fig. S8, ESI†). In addition, we also performed computational studies to identify the binding interactions among the surfactant and ATP, ADP, G6P, Ade and Pi using the software GAUSSIAN.^58,59^ We theoretically calculated the thermodynamic parameters (Δ*G* and Δ*H*) between the headgroup of the surfactant and ATP, ADP and G6P, separately (Fig. S9–S14 and Tables S2–S8, ESI†). This study provides a qualitative idea of the binding pattern, which follows the order ATP > G6P > ADP > Pi > Ade. It is worth noting that these calculations considered only the interaction between the surfactant headgroups and the nucleotides, which are far simpler than the multivalent interactions between the surfactant molecules with a long hydrocarbon chain and the phosphates, which were not considered in this study. The bond lengths between the atoms provided additional evidence in this regard (Fig. S9–S14, ESI†). The zeta potential (*ζ*) values also suggested that the binding of ATP with the surfactant is stronger than that of ADP + G6P, as the *ζ*-value of 50 μM of the cationic micellar assembly changed from 42 ± 4 mV to 24 ± 3 mV or 30 ± 2.5 mV after the addition of 10 μM of ATP or (ADP + G6P), respectively (Fig. S15 and S16, ESI†).

**Fig. 3:**
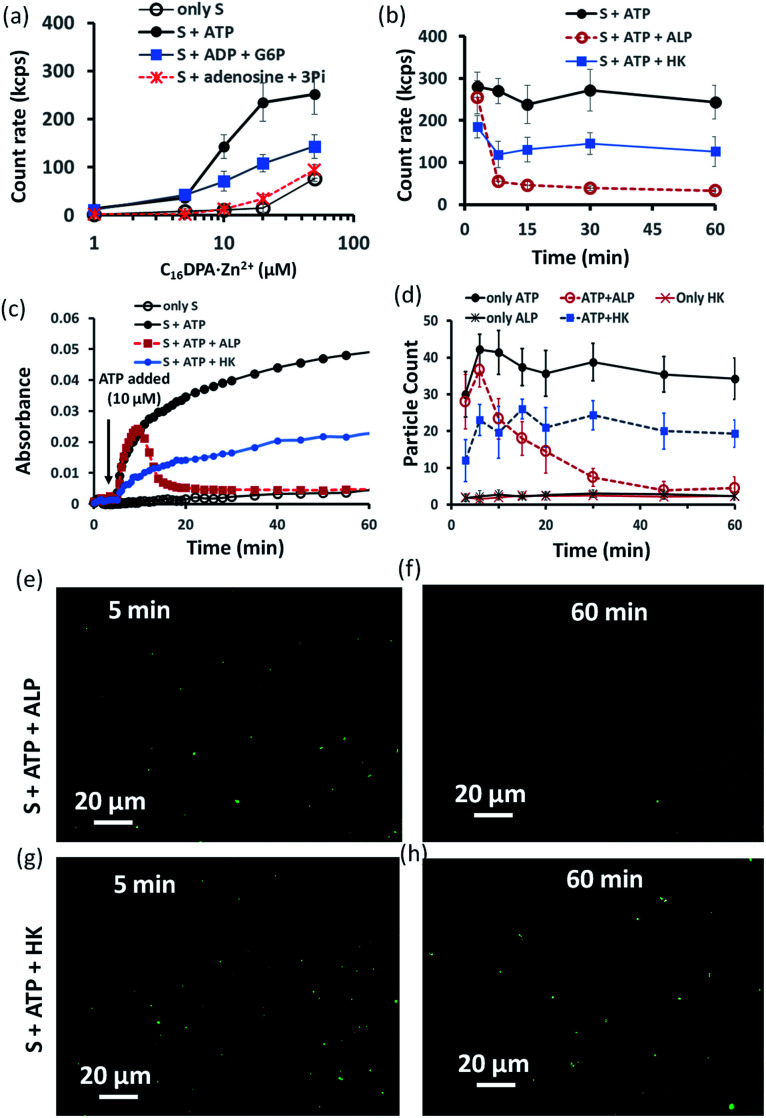
(a) Scattering intensity rate (count rate, kcps) of the formed aggregates as a function of the C_16_DPA⋅Zn^2+^ concentration in absence and presence of ATP (10 μM), ADP (10 μM) + G6P (10 μM) and Ade (10 μM) + Pi (30 μM) measured using dynamic light scattering (DLS). (b) Scattering intensity rate (count rate, kcps) measured by dynamic light scattering (DLS) of the aggregates as a function of time at fixed C_16_DPA⋅Zn^2+^ concentration (25 μM) and either presence of ALP (100 nM) or HK (50 nM) after addition of ATP (10 μM). (c) UV-vis absorbance value (at 600 nm) as a function of time after adding ATP (10 μM) when the system consists of either ALP (100 nM) or HK (50 nM). (d) Statistical analysis (in terms of the number of fluorescent dots) of the images taken at different time intervals after inserting the mixture solution of the surfactant (25 μM) in absence or presence of ATP (10 μM) and enzymes in a secure-seal hybridization chamber attached in a glass slide over a period of 1 h. (e–h) Representative images of the reaction mixture after 5 and 60 min after addition of C_16_DPA⋅Zn^2+^ (25 μM) and ATP (10 μM) in presence of either ALP (100 nM) or HK (50 nM) and C153 (2.5 μM) as the fluorescence dye. Experimental conditions: [HEPES] = 5 mM, [Glucose] = 1 mM, [Mg(NO_3_)_2_] = 0.5 mM, pH 7.0, *T* = 25 °C.

Indeed, we found that in the presence of ATP, the assembly formation starts at a concentration almost 3–4 times lower than the CAC of the surfactant, as the count rate in DLS started to increase after adding only 5–10 μM of C_16_DPA·Zn^2+^. Interestingly, the count rate also showed an increase, but at a lower rate compared to that for ATP, upon the addition of surfactant to a system containing 10 μM of ADP and G6P, whereas in the presence of Ade (10 μM) and Pi (30 μM), the count rate increase was almost the same as that for the surfactant alone (Fig. 3a). We also observed the ATP-bound aggregates using the transmission electron microscopy (TEM) technique, and the sizes of the aggregates (200 ± 50 nm) were comparable to those obtained using DLS (Fig. S17 and S18, ESI†). However, the previous experiment regarding the DLS count rate indicates that the introduction of HK into the surfactant system can indeed cause a different kind of assembly behavior, in which the assembly is initially mediated by ATP, but over time, the assemblies remain stabilized by ADP and G6P. In fact, we were pleased to observe that in the presence of ALP, the assemblies initially formed but disappeared within 15 min, whereas in the case of HK, there is little decay in the count rate or scattering for up to 1 h (Fig. 3b). The different temporal self-assembly responses are attributed to the multivalency effect of the different end products generated due to the action of the different enzymes (ALP or HK) towards the same substrate (ATP). We saw similar behavior in which the count rate measured from DLS decreased with decrease in size of the aggregate and eventually returned almost to the level observed for the surfactant alone (with no proper data and a very low count rate) after 60 min for the ALP-containing system, whereas this value remained almost constant for the HK-containing system (Fig. S17a–d, ESI†). Notably, in the absence of enzymes and the presence of ATP, the DLS count rate and size remain almost unchanged (Fig. 3b and S17e, ESI†).

Similar behavior was also observed for the turbidity due to the formation of larger-sized colloidal assemblies (measured by following the UV-vis absorbance at 600 nm) in the system upon the addition of ATP when either ALP or HK was already present in the system along with glucose and Mg^2+^ (Fig. 3c). Here, the control experiment with only the surfactant and enzyme did not result in any increase in the count rate or turbidity (Fig. S19, ESI†). We then used the hydrophobic fluorescent probe coumarin 153 (C153) to visualize the time-dependent assembly formation under a fluorescence microscope.^28^ Fluorescent particles appeared upon the introduction of ATP when no enzyme was used and their number remained almost constant over time, whereas the particles disappeared within 20 min in the case of ALP, and the number of particles formed in the case of HK was lower but remained constant. Control experiments with only the surfactant and surfactant + enzymes did not result in any significant number of fluorescent particles (Fig. 3d–h and S20–S28, ESI†). Overall, these experiments suggest that the agonistic conditions generated due to the action of the HK enzyme (comparable to case II and III in Fig. 1b) can sustain the assembly formation, unlike ALP, which acts in an antagonistic manner (comparable to case I of Fig. 1b) in terms of maintaining the structure of the aggregates.

**Fig. 4:**
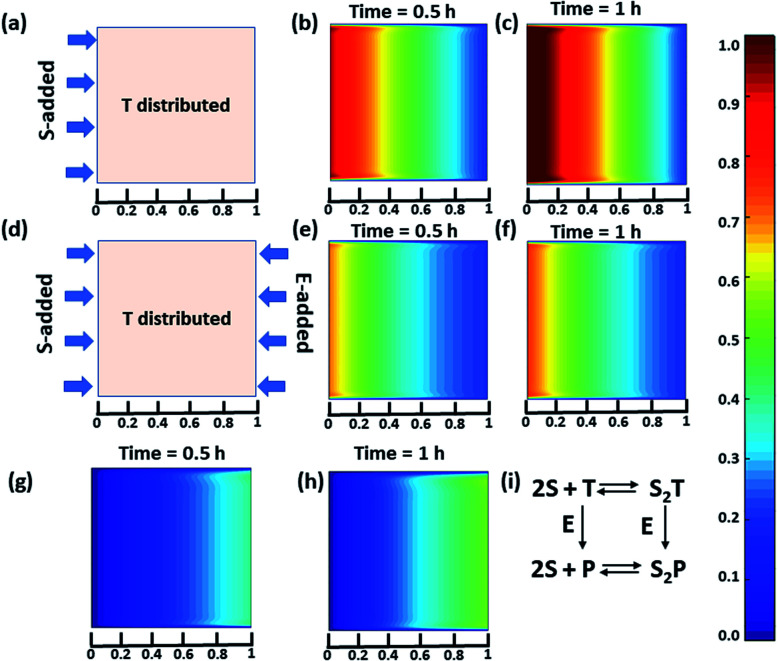
(a) Representative grid space when T is homogeneously distributed in space and S is added from the left boundary. Compositional distribution of S_2_T (b) 0.5 h and (c) 1 h after adding S from the left boundary. (d) Representative sample space when T is distributed evenly and S and E are added from the left and right boundaries, respectively. S_2_T distribution after (e) 0.5 h and (f) 1 h, and S_2_P compositional distribution (g) 0.5 h and (h) 1 h after the addition of S and E from the left and right boundaries, respectively. (i) Set of equations followed in presence of E (for details see ESI†). Time is a dimensionless unit in this system; however, for the sake of logical comparison with the experiment, the hour has been chosen as the unit of time.

Next, we were curious to observe the spatiotemporal assembly behavior when concentration gradients of the surfactant and enzymes were introduced into the system from opposite sides. This part will be of importance for generating dynamic surface patterns that can be controlled by enzymatic processes.^13,60–63^ For this, we first explored the emerging behavior under flow conditions *via* a computational model using MATLAB R2019b, FEATool Multiphysics (Fig. 4 and S29–S31, see ESI† for details).^60,61^ Here, we collectively studied the transport mechanism of both the reactive and non-reactive species involved in the system, along with their rates of formation and dissociation (the mass balance equation described in eqn (S1)–(S4)† has been employed, which was used for Fig. 1) using computational fluid dynamics. Here, Fick's second law was used to obtain the distribution of each species involved as functions of time and space in the two-dimensional system. We selected a square-shaped space throughout which T (which helps to form the dimerized assembly of the monomer S) was homogeneously distributed. From the left side, the monomer S, and from the right side, the enzymatically formed P, then decreased in a periodic manner to simultaneously form S_2_T and S_2_P in the square-shaped space. We assumed that monomer S and E do not interact, and thus, we did not use any E in the system; instead, only P (enzymatically transformed T) was considered. The colour scale ranging from red to blue denotes the relative concentration of the assembled species (either S_2_T or S_2_P) in the different zones. First, we analysed the distribution of S_2_T in the absence of any product gradient with the addition of S from the left side. In this case, S_2_T reached about 60% of the maximum possible intensity in terms of distribution at the exact midpoint after 1 h. However, when the enzyme was also added from the opposite side, the amount of S_2_T was almost 30% lower at the left side near the S-rich zone compared to that in the system without the enzyme, and at the midpoint, it reached ∼40% of the maximum possible intensity. Interestingly, in this case, the enzyme can convert T to P, which also has the ability to form S_2_P. Therefore, we also observed the formation of S_2_P with 40% of the maximum possible distribution near the right-side boundary where the enzyme was added. We also analysed a few other possible scenarios using the same system for which the rate of change of S_2_P at the right-side boundary is different (Fig. S31, ESI†). Additionally, a detailed zone-wise analysis of the theoretical outcome of the spatiotemporal differentiation of the S_2_T and S_2_P intensity distribution has been given in the ESI for the case described in Fig. 4 (Fig. S32 and S33, ESI†). Overall, this analysis showed that the distribution of the assemblies on a 2-D surface can be modulated over time using enzymes, and variable surface patterns can be obtained in this manner.

**Fig. 5:**
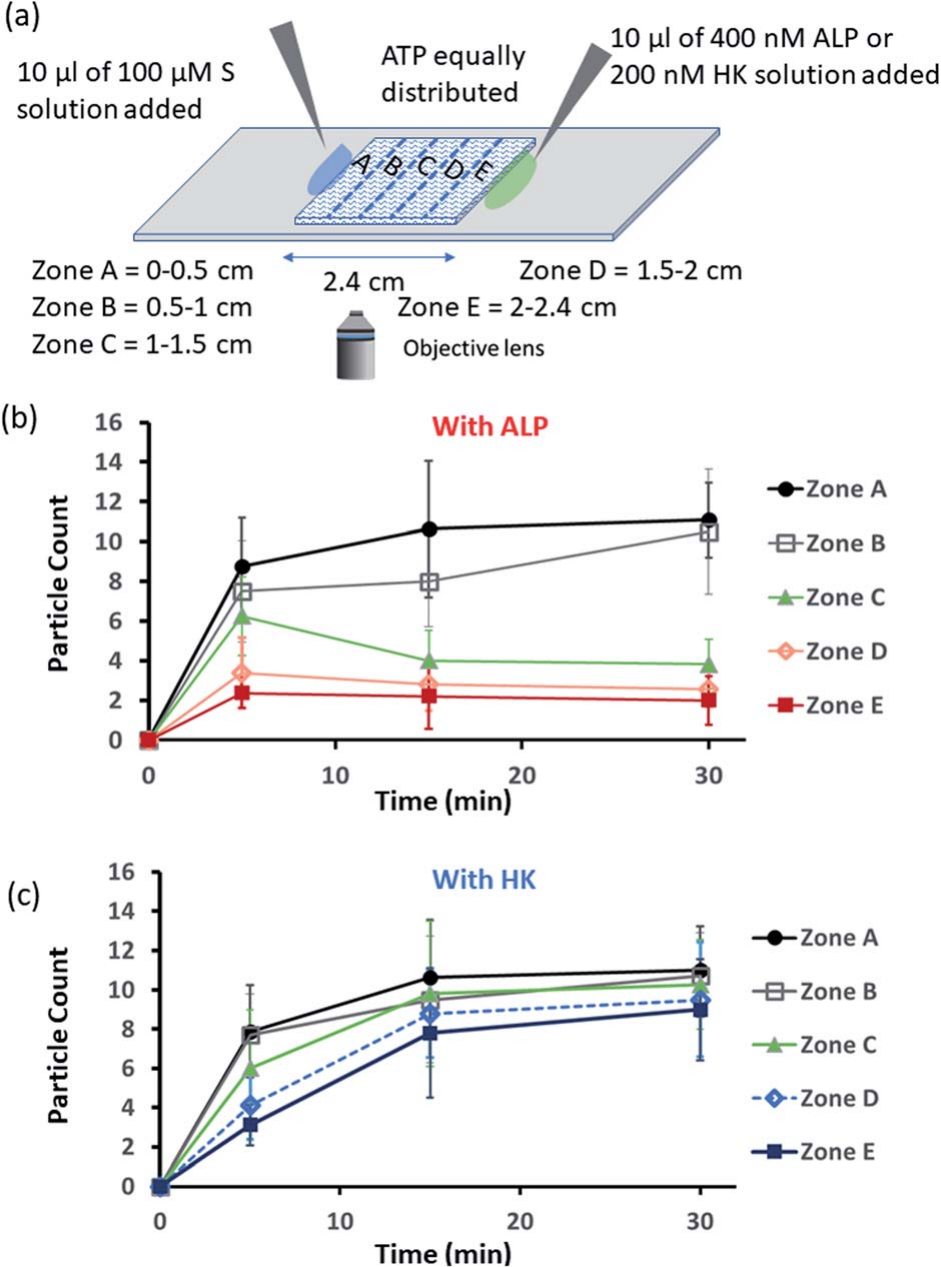
(a) Schematic representation of the formation and evolution of fluorescent structures over time in a space having a concentration gradient of the surfactant (S), C_16_DPA·Zn^2+^, from the left side and of the enzyme (either ALP or HK) from the right side, and in which ATP and glucose and Mg(NO_3_)_2_ have been homogeneously distributed. Please see the ESI† for details of the experimental protocol. The experiments were performed by placing a square-shaped glass coverslip (2.4 cm × 2.4 cm) on a glass slide on which there was already a drop (20 μl) of ATP (20 μM) mixed with C153 (2.5 μM). The area under the coverslip was divided into five parts, and images were taken under a fluorescence microscope. (b and c) Absolute count of the aggregated fluorescent structures evolved in the different zones (A–E) after 5 and 30 min when ALP or HK was added from the right side. Images were taken under the fluorescence microscope at five different positions in each zone at 40× zoom, and the experiments were replicated three times to obtain an average of 30 images per zone.

Encouraged by the theoretical results, we were eager to observe whether the assembled structures could be grown in spatially separated zones having concentration gradients of both the substrates and the enzymes (ALP and HK) at two opposite sides (Fig. 5a). For this purpose, we used a glass slide with 20 μl of 20 μM ATP (also containing 2.5 μM of C153 for microscopic visualization) on it.^41^ A cover slip (2.4 cm × 2.4 cm) was placed over the ATP solution, and at two opposite edges, 10 μl of a 100 μM C_16_DPA·Zn^2+^ solution and 10 μl of a 400 nM ALP or 200 nM HK solution were added to create concentration gradients on the opposite sides (details in Fig. S34, ESI†). The area under the glass cover slip was divided into five zones A–E as depicted in Fig. 5a. We then observed how the fluorescent structures evolved in these five zones over time. We were able to follow the number of structures formed in the different zones for up to 30 min, after which the slides dried out. We observed that in the absence of any enzyme, after 5 min the number of fluorescent structures was higher in zones A and B than in zones C–E, which are far from the surfactant addition area. However, after 15 and 30 min, the numbers of structures in zones C–E increased considerably, and the rate of increase in the fluorescence structures in all the zones remained almost equal (Fig. S35 and S36, ESI†). In this case, the statistical significance in terms of obtaining the fluorescent structures in neighbouring zones were also not distinctive, as the *p*-value was on the higher side (Tables S9 and S10, ESI†). Interestingly, when ALP was added at the side opposite the surfactant, the fluorescent structures changed dynamically with time, and in particular, a sharp change was observed in zone C at 15 min (Fig. 5b and S37, ESI†). The statistical significance of the difference between the B and C zones is clear (*p* value < 0.0001) at 15 and 30 min (Tables S11 and S12†). In this case, the number of structures is much higher in zone A and B *i.e.*, the surfactant addition zone, compared to zone C–E. On the contrary, the addition of HK results in spatiotemporal assembly behavior that is rather similar to that observed without enzymes, with a slightly higher number of structures in zones D and E (Fig. 5c and S38, ESI,† for *p*-values see Tables S13 and S14 in the ESI†). In this case, we observed a convergence in the number of structures in the zones with time. Thus, we have shown that the utilization of this strategy results in the design of three different types of spatiotemporally distinct dynamic surface patterns: (i) using only a gradient of the surfactant from one side, an almost parallel spatiotemporal increase in the number of structures between zones was observed from 5 to 30 min; (ii) using gradients of the surfactant and ALP at opposite sides, ALP works antagonistically to create a divergence and distinct zones of structural distribution (in our case, zones A and B are enriched with fluorescent structures, while zones C–E have a lower number of structures); and (iii) using gradients of the surfactant and HK at opposite sides, HK works agonistically and tends to create convergence among the zones in terms of the population density of fluorescent structures.

We also carried out a zone-wise comparison of the results obtained from the theoretical prediction and experimental data. For the theoretical points, we plotted the number corresponding to the color intensity and also divided the 2-D space into five equal zones A–E along the *x*-axis to provide a similar analysis for comparison with the experimental results (Fig. S39–S41, ESI†). We observed similar trends in the increase in the number of S_2_T assemblies (theoretical) and surfactant-bound ATP aggregates (experimental) in the absence of any enzyme, *i.e.*, when only S or the surfactant was added at the left side (please compare Fig. S32 and S35, ESI†). Also, similar trends of divergence and convergence of the structure population density with time from zone A to E in the presence of the enzymes ALP and HK was observed in both the theoretical and experimental studies (for comparison of ALP, please see Fig. S33b† for the theoretical results and Fig. 5b for the experimental ones; and for HK, please see Fig. S33d† for the theoretical results and Fig. 5c for the experimental pattern). For further clarification, we also compared zones A–E in the experiment at 30 min to the fluorescent structures and color intensity points at 1 h for all three cases as discussed above (Fig. S39–S41, ESI†).

**Fig. 6:**
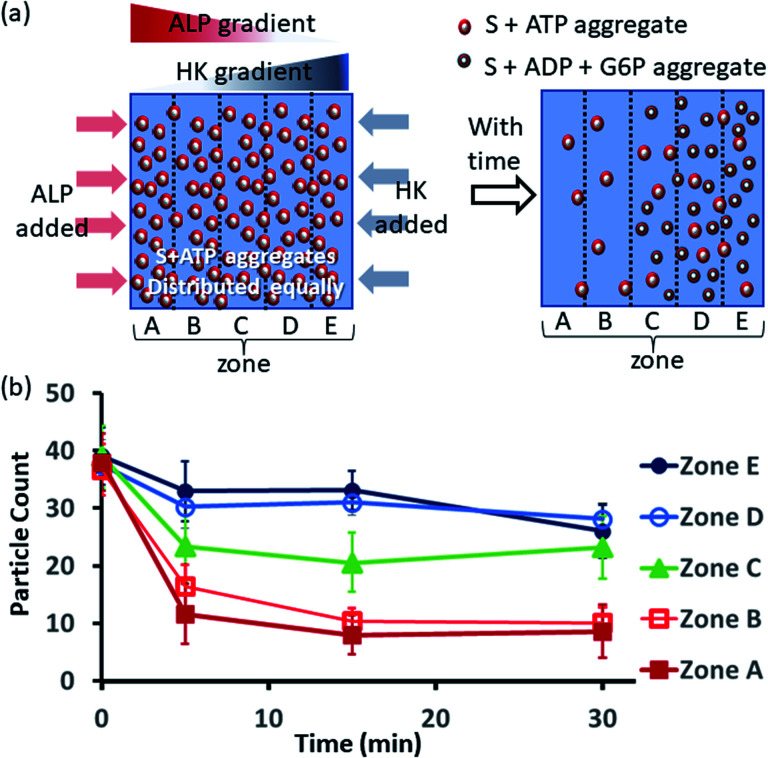
(a) Schematic representation of the formation and evolution of fluorescent structures over time in a space having concentration gradients of ALP and HK from the left and right sides, respectively, in which a pre-aggregated surfactant (S) + ATP mixture was homogeneously distributed. Please see the ESI† for details of the experimental protocol. The experiments were performed by placing a square-shaped glass coverslip (2.4 cm × 2.4 cm) on a glass slide with a 20 μl drop of pre-aggregated surfactant (50 μM) + ATP (20 μM) mixed with C153 (2.5 μM), glucose (2 mM) and Mg(NO_3_)_2_ (1 mM). The area under the coverslip was divided into five parts and images were taken under a fluorescence microscope. (b) Absolute count of the aggregated fluorescent structures evolved in the different zones (A–E) after 5, 15 and 30 min when ALP or HK were added at the left and right sides. Images were taken under a fluorescence microscope at five different positions in each zone at 40× zoom, and the experiments were replicated three times to obtain an average of 30 images per zone.

Next, we also performed an experiment in which we kept surfactant + ATP aggregates under a coverslip to obtain a uniform distribution. We then added the enzyme ALP at the left side and the enzyme HK at the right side to create antagonistic conditions on the left and agonistic conditions on the right (Fig. 6a and S42, ESI†). We monitored the changes in the populations of structures from zones A to E as described in the preceding paragraph after 5-, 15- and 30 min time intervals (Fig. 6b, S43 and S44, ESI†). We observed that the density of aggregates decreased over time near the ALP addition side, with a decrease of almost 70–80% after 15 min in zones A and B. On the contrary, near the HK addition side (zones D and E), the structural population remained almost constant up to 15 min, and only a ∼20% decrease was observed at 30 min, presumably due to the formation of considerably higher amounts of ADP and G6P, which have a slightly lower aggregate formation ability than ATP (please see also Fig. 3a–d). Here, distinct differences were also observed between zones A and B with a low fluorescent structure population density and zones C to E with a higher population density of fluorescent structures.

## Conclusions

We have shown that judicious exploitation of enzymes can create different spatiotemporal dynamic self-assembly behaviours. Unlike previous reports of transient self-assembly or self-assembly under dissipative conditions,^37,40,64^ here, we have shown both theoretically and experimentally that it is also possible to sustain the assembly behavior and generate a surface pattern based on the spatiotemporal modulation of the population density of the aggregates. In fact, spatiotemporal organization can be programmed, and the utilization of this chemistry may potentially lead to more diverse types of complex surface patterns. It is to be noted here that this type of reaction–diffusion system has been generated in previous reports in the literature using inorganic salts and their reactions to create a range of patterns, which contributed to the advancement of dynamic micro- and nanotechnology and autonomous devices.^65–68^ In addition, recent reports have demonstrated spatiotemporal ordering and reaction networking in supramolecular gel networks using organic reaction-, pH- or enzyme-reaction-based systems.^69–73^ Herein, we have shown the assembly formation dynamics between a surfactant and nucleotides/phosphosugars. Several non-equilibrium organizational patterns can be obtained depending on the choice of enzyme, and the patterns are distinctly different if no enzyme is used. In this manuscript, we have shown three different cases mediated by enzymes: (i) a gradient of agonistic conditions, (ii) a gradient of antagonistic conditions, and (iii) simultaneous agonistic and antagonistic gradients at two opposite sides. As the population of self-assembling units can be organized at will, it is also potentially possible to control multiple reactions or other types of functionality selectively at precise locations that are activated either in the assembled or disassembled state to generate multilevel spatiotemporal control over multiple functions, and this approach could thus play an important role in designing life-like autonomous and intelligent systems.^13,68^ Overall, we believe that these results are important for the development of nonlinear dynamical systems with complicated life-like properties that emerge from relatively low-complexity or quite simple systems.

## Experimental section

### Materials and methods

All commercially available reagents were used as received without any further purification. The chemicals sodium phosphate dibasic (Na_2_HPO_4_), hexadecyl bromide (C_16_H_33_Br), and adenosine were procured from Sisco Research Laboratory (SRL), India. Adenosine 5′ triphosphate disodium salt (ATP), adenosine 5′ diphosphate disodium salt (ADP), di-picolyl amine, and alkaline phosphatase of bovine origin were purchased from Sigma-Aldrich. The chemicals potassium carbonate (K_2_CO_3_), glucose (C_6_H_12_O_6_), potassium iodide (KI), and coumarin 153 were also purchased from SRL India, and magnesium nitrate was obtained from Molychem. Glucose-6-phosphate (G6P) and hexokinase were procured from Hi media. HPLC grade methanol and acetonitrile purchased from Sigma-Aldrich were used in synthesis and HPLC.

UV-vis studies were performed using a Varian Cary 60 (Agilent Technologies) spectrophotometer. The total reaction volume in the cuvette was fixed at 1 ml, and a cuvette with a path length of 1 cm was used for the entire kinetic study. All measurements were performed at 25 °C.

The NMR spectra were recorded using a Bruker Avance-III 400 MHz spectrometer. ^1^H and ^13^C NMR spectra were recorded at operation frequencies of 400 MHz and 100 MHz, respectively. CDCl_3_ and D_2_O were used as the solvents and tetramethylsilane (TMS) was used as the internal standard for recording the samples. The chemical shift values are reported as delta (*δ*) units in parts per million (ppm).

HRMS was recorded using Waters Synapt G2-Si Q ToF Mass Spectrometer.

The fluorescence microscopic images were collected using a Zeiss Axis Observer 7 microscope with an AxioCam 503 Mono 3 Mega pixel with ZEN 2 software.

The transmission electron microscopy images were taken using a JEOL JEM-F200 microscope.

The dynamic light scattering (DLS) data were recorded using a Malvern Zetasizer Nano-ZS90.

HPLC spectra were recorded using an Agilent 1260 Infinity II.

### Synthesis of the C_16_DPA·Zn^2+^

C_16_DPA was synthesized according to a literature protocol.^74^ Di-(2-picolyl)amine(100 mg, 0.5 mmol) and 1-bromohexadecane were added to a suspension of K_2_CO_3_ in MeCN (10 ml). The suspension was refluxed and stirred for 24 h, after which it was filtered. After evaporation under reduced pressure, the crude product was purified using column chromatography (silica gel, eluent: hexane/EtOAc) yielded the product as a faintly yellow sticky oil (70%).

C_16_DPA (80 mg, 0.19 mmol) and Zn(NO_3_)_2_ (35.97 mg, 0.19 mmol) were added to a round bottom flask and the reaction mixture was refluxed in MeOH for 3 h. The mixture was then evaporated to dryness under vacuum in a rotatory evaporator yielding the product as a faint yellow solid, which was used for further observations without any purification.

^1^H NMR (*δ* ppm, CDCl_3_, 298 K, 400 MHz): 8.53 (d, *J* = 4 Hz, 2H), 7.67 (td, *J* = 8, 8 Hz, 2H), 7.57 (d, *J* = 8 Hz, 2H), 7.17–7.14 (m, 2H), 3.82 (s, 4H), 2.56–2.53 (m, 2H), 1.54 (m, 2H), 1.25 (m, 26H), 0.90 (t, *J* = 4 Hz, 3H).

^13^C NMR (*δ* ppm, CDCl_3_, 298 K, 100 MHz): 160.19, 148.91, 136.35, 122.81, 121.82, 60.50, 54.53, 31.93, 29.70, 29.67, 29.64, 29.49, 29.37, 27.33, 27.09, 22.70, 14.13.

ESI-MS (ESI+, H_2_O) [M + H]^+^: found: 424.3627; calculated: 424.3613.

The NMR (^1^H, ^13^C) and ESI-MS spectra are shown in ESI† Fig. S2–S4.

### Counting of fluorescent particles

Counting of fluorescent particles was carried out using the software imageJ. For each frame, first, the background noise was cleared using the software tool. The resulting image was converted to an 8-bit image and also set to binary mode. Subsequently, the threshold was adjusted and the image was then analysed to obtain the number of particles for each image.

## Data availability

See ESI† for complete synthetic procedures, numerical analysis, DFT data, additional DLS, zeta potential, HPLC, and turbidity data, fluorescence microscopy and TEM images, and the MATLAB study for theoretical analysis.

## Author contributions

Priyanka carried out the synthesis and most of the experimental part. E. S. carried out part of the experiments, along with the numerical modelling and MATLAB study. S. K. B. carried out the Gaussian-related calculations. R. R. M. carried out part of the experiments. S. M. conceived the idea, designed the experiments and wrote the manuscript, and all the authors commented on it.

## Conflicts of interest

There are no conflicts to declare.

## Supplementary Material

SC-013-D1SC05353A-s001

SC-013-D1SC05353A-s002

SC-013-D1SC05353A-s003

SC-013-D1SC05353A-s004
