# Supplementary material for: Spatiotemporal dynamics of self-assembled structures in enzymatically induced agonistic and antagonistic conditions
Source: Chem Sci. 2021 Nov 22;13(1):274–82. doi: 10.1039/d1sc05353a (PMC8694342; doi:10.1039/d1sc05353a)
Supplement: SC-013-D1SC05353A-s001 [file SC-013-D1SC05353A-s001.pdf]

Electronic Supplementary Material (ESI) for Chemical Science.
This journal is © The Royal Society of Chemistry 2021

# Spatiotemporal Dynamics of Self-assembled Structures in Enzymatically Induced Agonistic and Antagonistic Conditions

Priyanka+, Ekta Shandilya+, Surinder Kaur Brar, Rishi Ram Mahato, Subhabrata Maiti*

+ These authors contributed equally

Department of Chemical Sciences, Indian Institute of Science Education and Research (IISER) Mohali, Knowledge City, Manauli 140306, India

## Electronic Supplementary Information

## Table of Contents

**A. Temporal evolution of assembly via numerical modelling ............................................ S2**

**B. Characterization of the synthesized surfactant, $C_{16}DPA{\cdot}Zn^{2+}$........................................ S6**

**C. Determination of the critical aggregation concentration (cac) of the metallo-surfactant $C_{16}DPA{\cdot}Zn^{2+}$ ......................................................................................................... S8**

**D. HPLC Analysis ...................................................................................................S10**

**E. DFT study .........................................................................................................S11**

**F. DLS and zeta potential study ..............................................................................S17**

**G. Transmission electronic microscopic (TEM) imaging ...............................................S20**

**H. Turbidity study ..................................................................................................S21**

**I. Fluorescence Microscopic study ..........................................................................S21**

**J. Spatiotemporal study of assembly formation using fluid dynamics ...............................S27**

**K. Experimental verification of spatiotemporal change in number of aggregates ...............S34**

**L. Supporting video..................................................................................................S45**

**M. References ........................................................................................................S46**

**A. Temporal evolution of assembly via numerical modelling**

Considering a system constituting a monomer S and chemical fuel T such that when two monomeric units of S comes in proximity of T to form a dimeric assembly $S_2T$ with a k1/k2 as equilibrium constant.

In addition to this, when we introduce an enzyme E to our system which can breakdown down T into P.

Now, coming to the enzymatic action, as E can cleave T to P, we now assume that it can cleave T from assembled state $S_2T$ with same rate constants as $k_{cat}$ (k) and $K_M$ according to Michaelis-Menten equation. Also, the product has some affinity towards S, such that it is also capable of forming dimeric assembly as $S_2P$. All the above-mentioned possibilities of assembly formation and deformation can be collectively represented by equations S1 -S4.

$$2S + T \underset{k_2}{\overset{k_1}{\rightleftharpoons}} S_2T \qquad (S1)$$

$$T + E \xrightarrow{k,\ k_M} P + E \qquad (S2)$$

$$S_2T + E \xrightarrow{k,\ k_M} 2S + P + E \qquad (S3)$$

$$2S + P \underset{k_4}{\overset{k_3}{\rightleftharpoons}} S_2P \qquad (S4)$$

Above coupled equations (S5-S9) can be represented by set of first-order differential equations formed according to mass-balance principle.

$$\frac{d[S]}{dt} = -2\left(k_1[S]_t^2[T]_t - k_2[S_2T]_t + k_3[S]_t^2[P]_t - k_4[S_2P]_t - \frac{k[E][S_2T]_t}{k_M+[S_2T]_t}\right) \quad (S5)$$

$$\frac{d[T]}{dt} = -k_1[S]_t^2[T]_t + k_2[S_2T]_t - \frac{k[E][T]_t}{k_M+[T]_t} \quad (S6)$$

$$\frac{d[S_2T]}{dt} = k_1[S]_t^2[T]_t - k_2[S_2T]_t - \frac{k[E][S_2T]_t}{k_M+[S_2T]_t} \quad (S7)$$

$$\frac{d[P]}{dt} = \frac{k[E][S_2T]_t}{k_M+[S_2T]_t} + \frac{k[E][T]_t}{k_M+[T]_t} - k_3[S]_t^2[P]_t + k_4[S_2P]_t \quad (S8)$$

$$\frac{d[S_2P]}{dt} = k_3[S]_t^2[P]_t - k_4[S_2P]_t \quad (S9)$$

To get compositional change of each component with respect to time the above-mentioned equations were solved using Python 3.0. To get the compositional change of individual component, the rate constants used in equation S1-S4 were fixed and so formed ordinary differential equations were solved using *odeint* function of Numpy packages [S1]. The code used for solving above-formed equations is shown in note S1. For instance, the parameters used are shown in table S1 and composition of dimeric assembly ($S_2T$ and $S_2P$) and total assembly formed ($S_2T$ + $S_2P$) is shown in figure S1. In addition to this, to get a broader view of effect of rate constants on individual components, parameters were varied (table S1) and the relative distribution of composition is shown in fig. S1.

It is worthy to note here time and concentration are dimensionless unit in this system, however, for the sake of logical comparison with experiment, hour has been chosen as the unit of time and µM as the unit of concentration respectively.

**Table S1:** Kinetic parameters used for numerical modelling for case I, case II, and case III.

| **Parameter (unit)** | **Case I** | **Case II** | **Case III** |
|---|---|---|---|
| $k_1$ ($\mu M^{-2}h^{-1}$) | 100 | 100 | 100 |
| $k_2$ ($h^{-1}$) | 0.01 | 0.01 | 0.01 |
| $k_3$ ($\mu M^{-2}h^{-1}$) | 0.0001 | 10000 | 0.01 |
| $k_4$ ($h^{-1}$) | 10000 | 0.0001 | 100 |
| k (kcat, $h^{-1}$) | 250 | 250 | 250 |
| $K_m$ (µM) | 50 | 50 | 50 |
| [Monomer][S, µM] | 200 | 200 | 200 |
| [Chemical fuel] ([T], µM) | 50 | 50 | 50 |
| [Enzyme] (E, µM) | 1 | 1 | 1 |

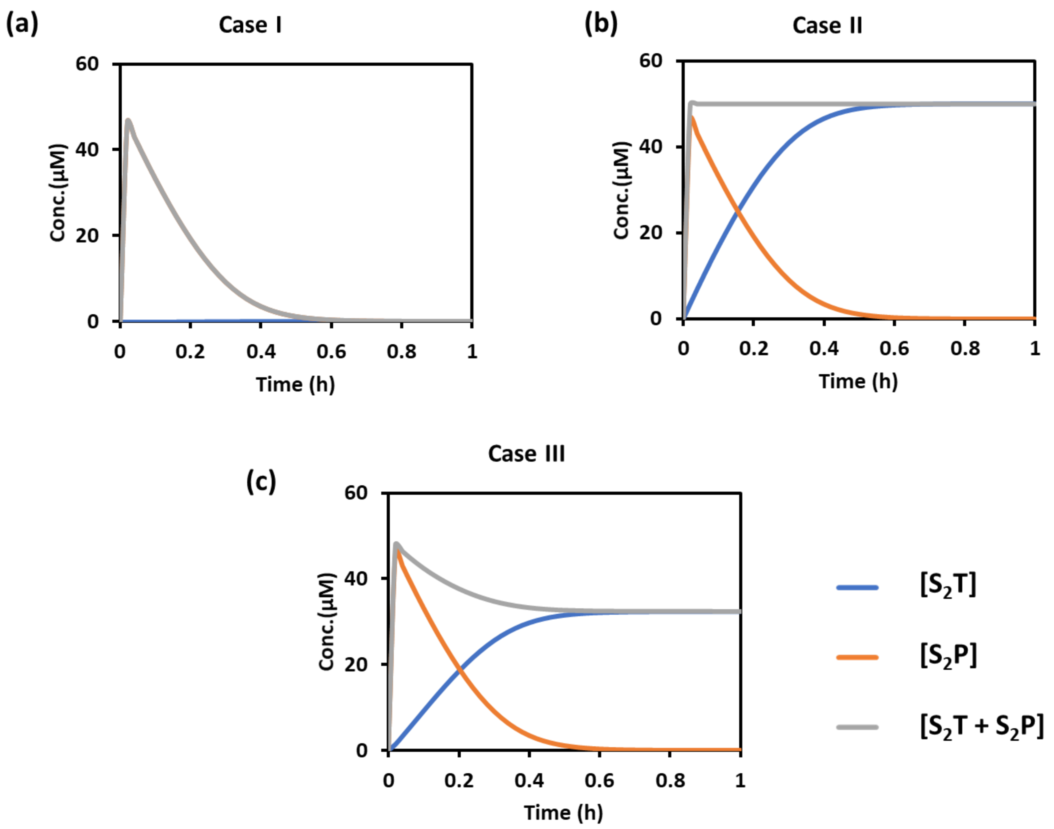


**Fig. S1.** Compositional change of dimeric assembly formation $S_2T$, $S_2P$, and sum of $S_2T$ and $S_2P$ over time derived from kinetic modelling. The kinetic parameters for case I, case II, and case III is shown in table S1.

Note S1:

```
#NoteS1


def rxn(z,t) :
  k = kcat*E
  r1 = k1 * z[0]*z[0]*z[1]
  r2 = k2 * z[2]
  r3 = k3 * z[0]*z[0]*z[3]
  r4 = k4 * z[4]
  ra = k*z[2]/(km + z[2])
  rb = k*z[1]/(km + z[1])

  dMdt = - 2 * r1 + 2 * r2 + 2 * ra - 2* r3 + 2 * r4 #S, MONOMER
  dWdt = - r1 + r2 - rb    #T, chemical fuel
  dUdt = r1 - r2 - ra      #S2T, dimeric assembly with T
  dNdt = ra + rb - r3 + r4    #P, product formed from T
  dVdt = -r4 + r3    #S2P, dimeric assembly with P
  return[dMdt,dWdt,dUdt,dNdt,dVdt]
```

## B. Characterization of the synthesized surfactant, $C_{16}DPA{\cdot}Zn^{2+}$

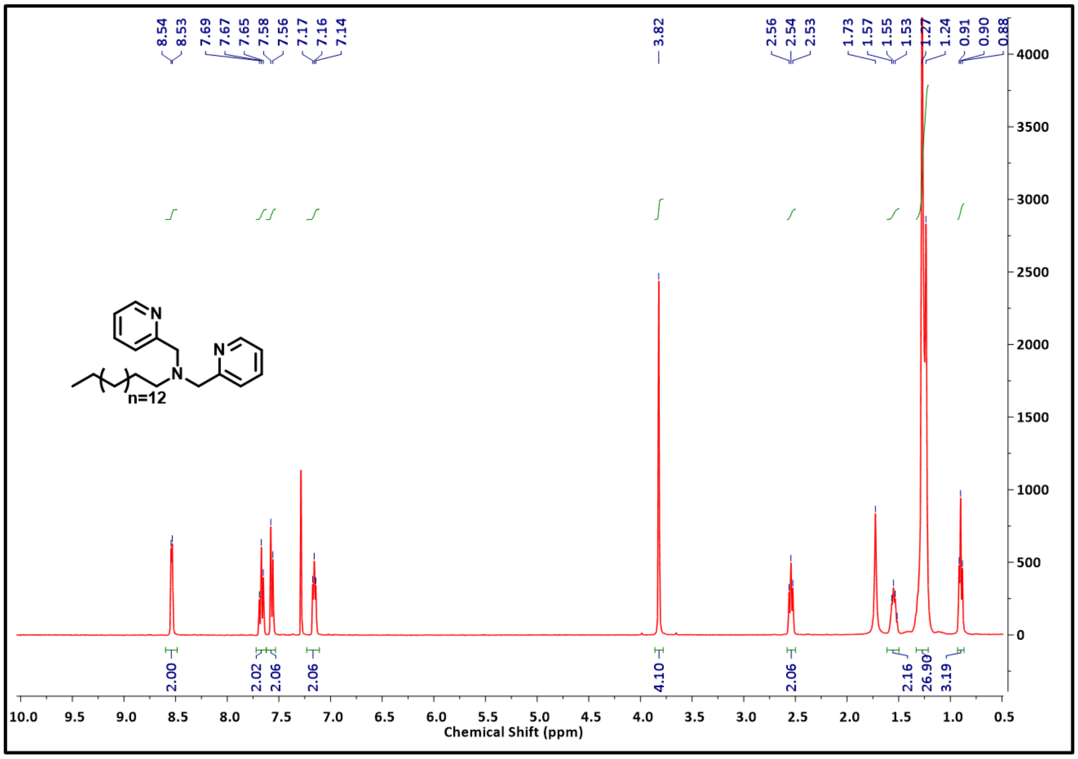


**Fig. S2.** $^1$H-NMR Spectrum of Compound $C_{16}DPA$ in $CDCl_3$.

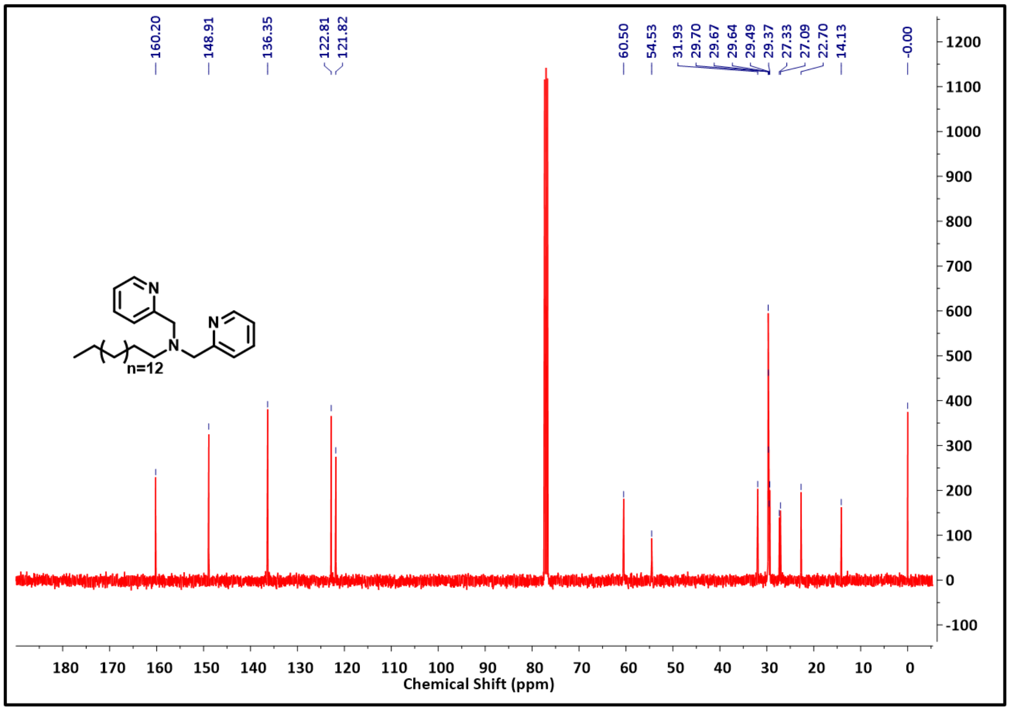


**Fig. S3.** $^{13}$C-NMR Spectrum of Compound $C_{16}$DPA in $CDCl_3$

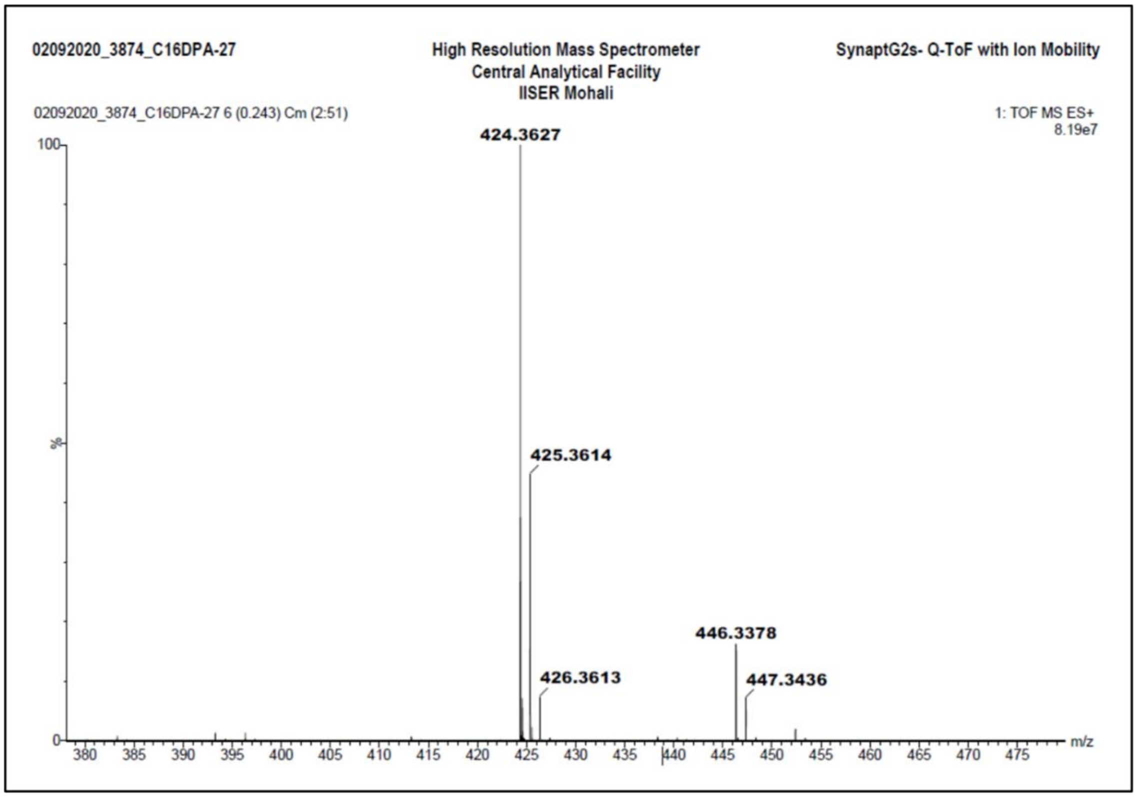


**Fig. S4.** HRMS of compound $C_{16}$DPA in H2O.

**C. Determination of the critical aggregation concentration (cac) of the metallo-surfactant $C_{16}DPA{\cdot}Zn^{2+}$**

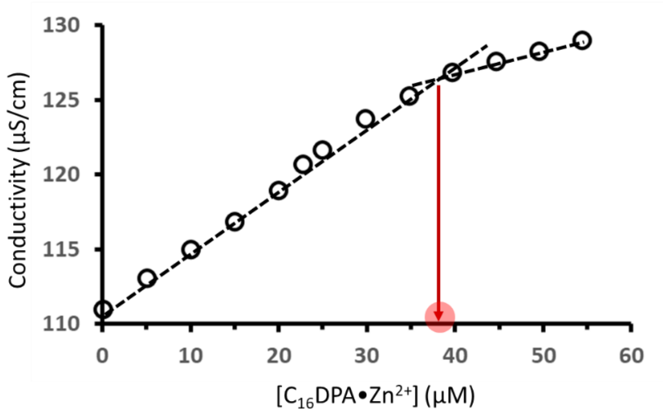


**Fig. S5.** Determination of the cac of surfactant $C_{16}DPA{\cdot}Zn^{2+}$ by conductivity. At 298 K, the specific conductance of surfactant in water was used to calculate the CAC. With a particular slope, the specific conductivity rises as the concentration of $C_{16}DPA.Zn^{2+}$ rises. The slope, however, varies depending on the concentration. The critical aggregation concentration is the point at which two linear fluctuations collide (CAC). Experimental conditions: [HEPES] = 5 mM buffer pH = 7, 25°C.

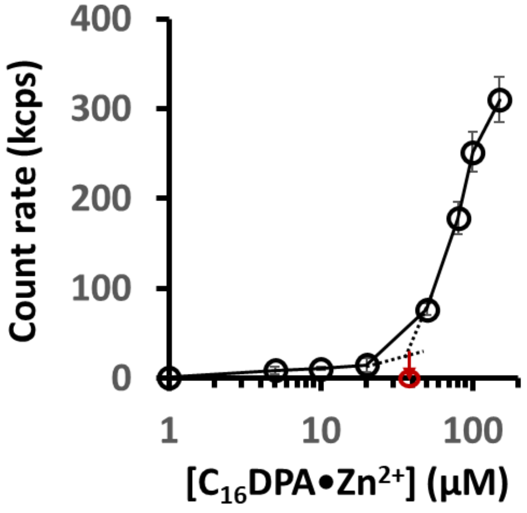


**Fig. S6.** Scattering intensity rate (count rate, kcps) measured by dynamic light scattering (DLS) of the aggregates formed as a function of $C_{16}DPA{\bullet}Zn^{2+}$ concentration. Experimental conditions: [HEPES] = 5 mM, pH 7.0, T = 25 °C. DLS count rate data also suggests that structure formation, thereby increase of count rate value starts around 35±5 µM, which implies CAC of the surfactant around 35±5 µM.

| | |
|---|---|
| Z-Average (d.nm): | 146.8 |
| PdI: | 0.132 |
| Intercept: | 0.971 |
| Result quality : | Good |

| | Size (d.nm): | % Intensity: | St Dev (d.nm): |
|---|---|---|---|
| Peak 1: | 168.8 | 100.0 | 67.49 |
| Peak 2: | 0.000 | 0.0 | 0.000 |
| Peak 3: | 0.000 | 0.0 | 0.000 |

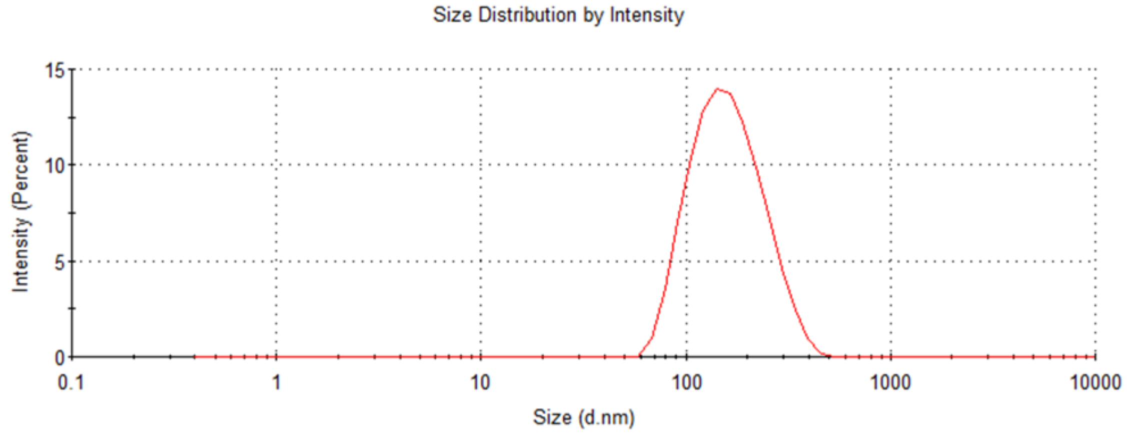


Size distribution by intensity

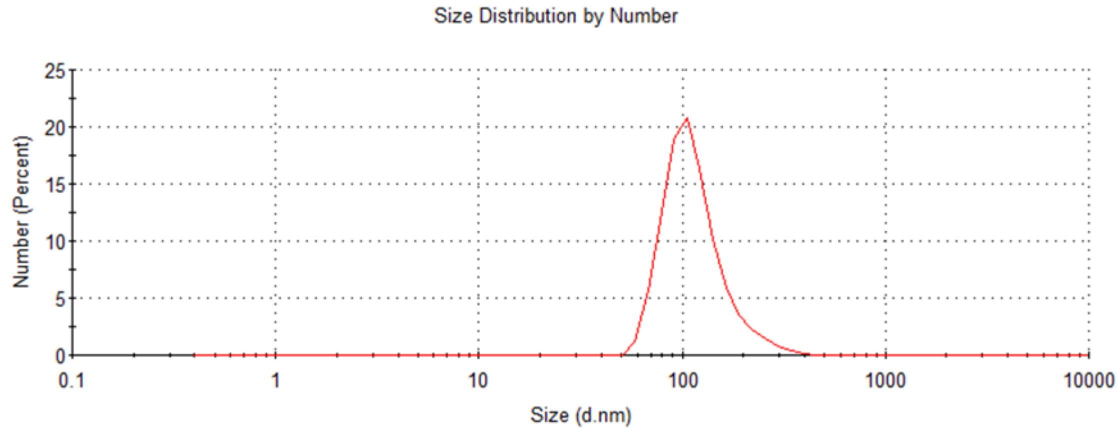


Size distribution by number

**Fig. S7.** DLS Size profile of [$C_{16}$DPA.$Zn^{2+}$ ]= 50 µM Experimental conditions: [HEPES]=5 mM buffer pH7, 25°C.

## D. HPLC Analysis

HPLC analysis were performed on an Agilent 1260 Infinity II apparatus and chromatographic separations were carried out on a C18 4µm, 150×4.6mm column. The mobile phase consists of A:150mM potassium phosphate buffer,pH7 and B:methanol (MeOH). The injection volume was 20µL and the system was run isocratically when ATP gets converted to Adenosine in presence of ALP at 20%B for 7 min, with flow rate of 0.6ml/min. Similarly the conversion of ATP to ADP in presence of HK was separated using 3% B for 7 min isocratically, with flow rate of 0.5ml/min and the column compartment was at 25°C. The detection wavelength was 259nm.

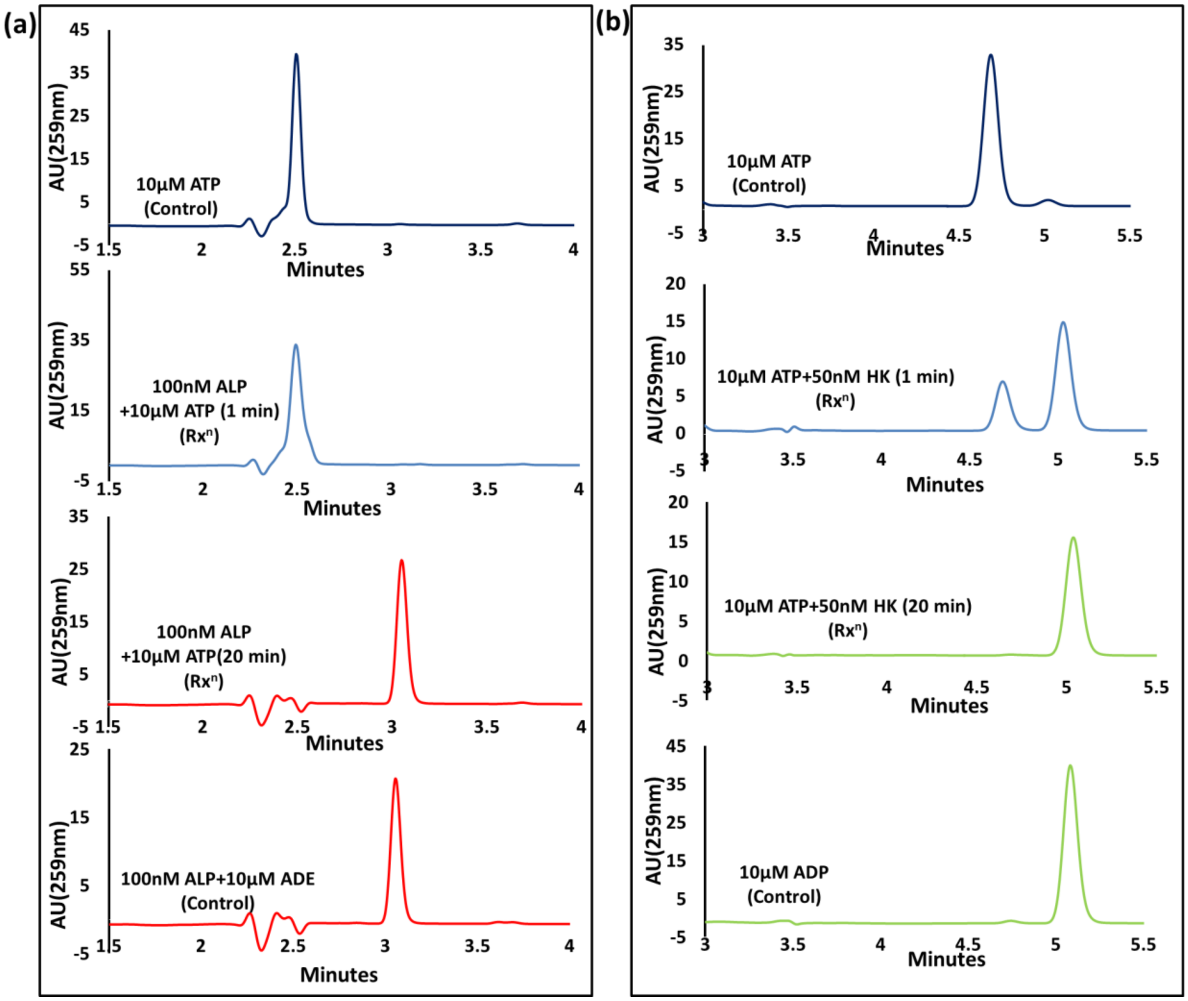


**Fig. S8.** HPLC Chromatogram of (a) ATP and Adenosine separated by C18 column in presence of ALP using phosphate buffer/MeOH (80:20; v/v) (b) Enzymatic conversion of ATP to ADP in presence of Hexokinase which was separated by C18 column using phosphate buffer/MeOH (97:3; v/v) Experimental conditions: [Glucose]= 1 mM, [$Mg(NO_3)_2$]= 0.5 mM, [HEPES]=5 mM buffer pH7, 25°C.

## E. DFT study

All quantum chemical calculations pertaining to surfactant and surfactant with ATP, ADP, G6P were performed by Density Functional Theory (DFT) method, using Gaussian 09 software.[S3] The geometries of stationary points were optimized by minimizing energies with respect to all geometrical parameters without imposing any molecular symmetry constraints using 6-311G basis set and for dispersion corrections, WB97XD, functional was used. Frequency calculations showed the absence of any imaginary frequency modes which confirmed that the optimized structure is an energy minimum. The solvent correction was done using conductor polarized continuum model (CPCM).

### Optimized geometries

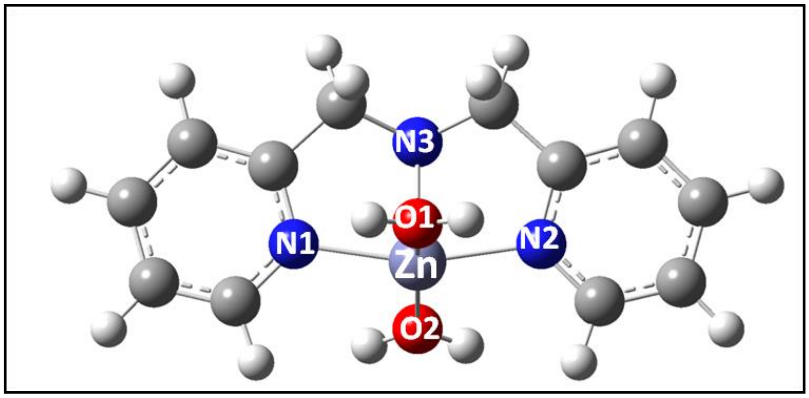


**Fig. S9.** Optimized molecular structure of DPA_$Zn^{2+}$ surfactant in solution calculated by 6-311G basis set using DFT/ WB97XD

**Table S2.** Calculated theoretical bond parameters of DPA_$Zn^{2+}$ surfactant using WB97XD/ 6-311G

| S. No. | Selected atoms | Bond distances (Å) |
|---|---|---|
| 1. | Zn-N1 | 2.06116 |
| 2. | Zn-N2 | 2.06116 |
| 3. | Zn-N3 | 2.12885 |
| 4. | Zn-O1 | 2.11931 |
| 5. | Zn-O2 | 2.05878 |

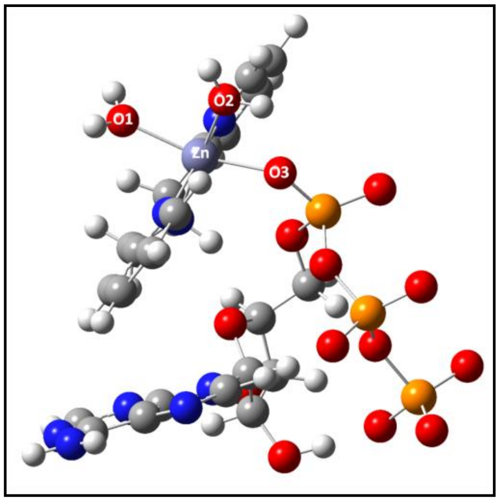


**Fig. S10.** Optimized molecular structure of DPA_$Zn^{2+}$ surfactant with ATP in solution calculated by 6-311G basis set using DFT/ WB97XD

**Table S3.** Calculated theoretical bond parameters of DPA_$Zn^{2+}$ surfactant with ATP using WB97XD/6-311G

| S. No. | Selected atoms | Bond distances (Å) |
|---|---|---|
| 1. | Zn-N1 | 2.09587 |
| 2. | Zn-N2 | 2.09487 |
| 3. | Zn-N3 | 2.14124 |
| 4. | Zn-O1 | 2.06395 |
| 5. | Zn-O2 | 2.15577 |
| 6. | Zn-O3 | 2.08641 |

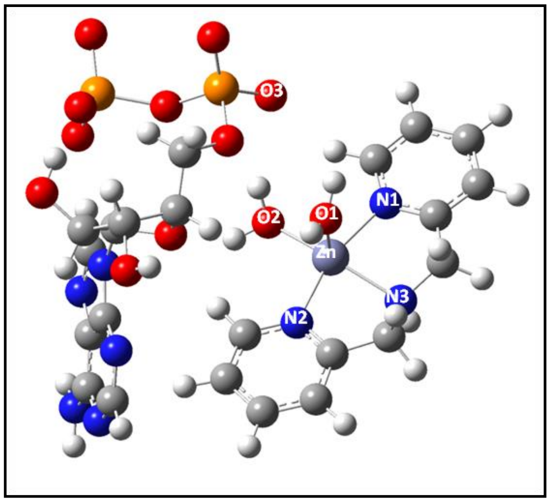


**Fig. S11.** Optimized molecular structure of DPA_$Zn^{2+}$ surfactant with ADP in solution calculated by 6-311G basis set using DFT/ WB97XD

**Table S4.** Calculated theoretical bond parameters of DPA_$Zn^{2+}$ surfactant with ADP using WB97XD/6-311G

| S. No. | Selected atoms | Bond distances (Å) |
|---|---|---|
| 1. | Zn-N1 | 2.07584 |
| 2. | Zn-N2 | 2.07179 |
| 3. | Zn-N3 | 2.13617 |
| 4. | Zn-O1 | 1.97905 |
| 5. | Zn-O2 | 2.13029 |
| 6. | Zn-O3 | 4.79354 |

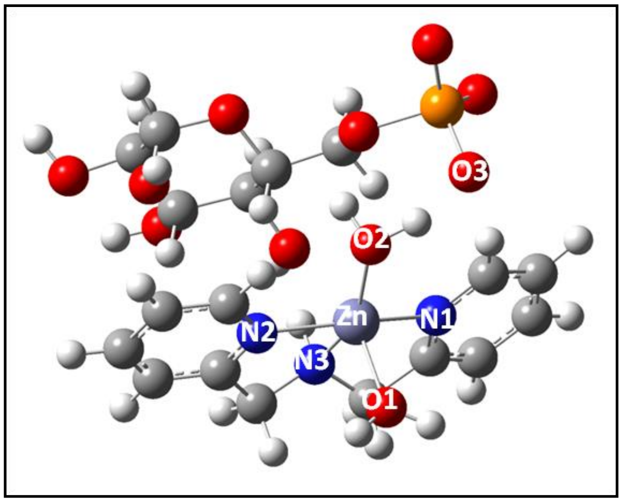


**Fig. S12.** Optimized molecular structure of DPA_Zn$^{2+}$ surfactant with G6P in solution calculated by 6-311G basis set using DFT/ WB97XD

**Table S5.** Calculated theoretical bond parameters of DPA_Zn$^{2+}$ surfactant with G6P using WB97XD/6-311G

| S. No. | Selected atoms | Bond distances (Å) |
|---|---|---|
| 1. | Zn-N1 | 2.08898 |
| 2. | Zn-N2 | 2.10916 |
| 3. | Zn-N3 | 2.12545 |
| 4. | Zn-O1 | 1.99382 |
| 5. | Zn-O2 | 2.06057 |
| 6. | Zn-O3 | 3.94602 |

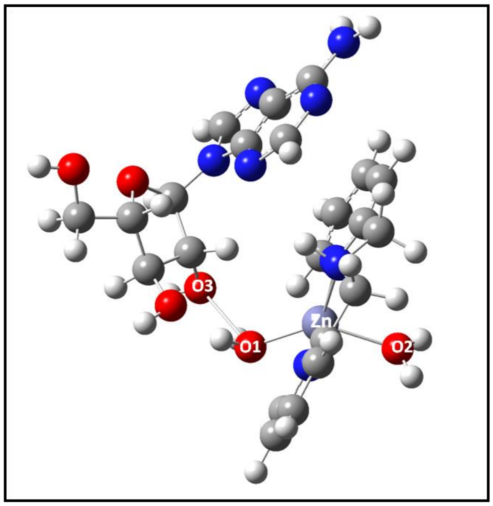


**Fig. S13.** Optimized molecular structure of DPA_$Zn^{2+}$ surfactant with adenosine in solution calculated by 6-311G basis set using DFT/ WB97XD

**Table S6.** Calculated theoretical bond parameters of DPA_$Zn^{2+}$ surfactant with adenosine using WB97XD/6-311G

| S. No. | Selected atoms | Bond distances (Å) |
|---|---|---|
| 1. | Zn-N1 | 2.09252 |
| 2. | Zn-N2 | 2.07583 |
| 3. | Zn-N3 | 2.14905 |
| 4. | Zn-O1 | 2.09372 |
| 5. | Zn-O2 | 2.01488 |
| 6. | O1-O3 | 2.64254 |

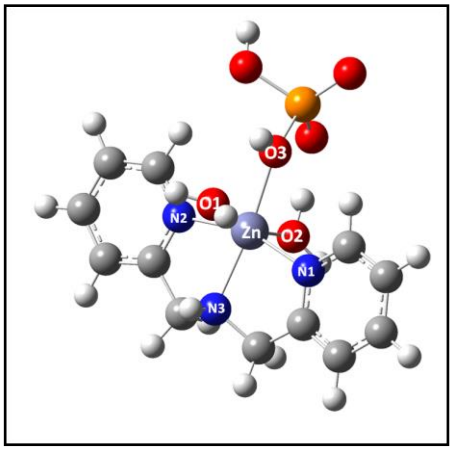


**Fig. S14.** Optimized molecular structure of DPA_$Zn^{2+}$ surfactant with $NaH_2PO_4$ in solution calculated by 6-311G basis set using DFT/ WB97XD

**Table S7.** Calculated theoretical bond parameters of DPA_$Zn^{2+}$ surfactant with $NaH_2PO_4$ using WB97XD/6-311G

| S. No. | Selected atoms | Bond distances (Å) |
|---|---|---|
| 1. | Zn-N1 | 2.07661 |
| 2. | Zn-N2 | 2.07657 |
| 3. | Zn-N3 | 2.14805 |
| 4. | Zn-O1 | 2.20990 |
| 5. | Zn-O2 | 2.08368 |
| 6. | Zn-O3 | 2.09390 |

**Table S8.** Comparison of theoretically calculated enthalpies (ΔH) and Gibbs Free Energies (ΔG) (in KJ/mol) for complexes DPA.$Zn^{2+}$/ATP, DPA.$Zn^{2+}$/ADP, DPA.$Zn^{2+}$/G6P DPA.$Zn^{2+}$/NaH2PO4, DPA.$Zn^{2+}$/Adenosine

| Complex | ΔH(KJ/mol) | ΔG(KJ/mol) |
|---|---|---|
| **DPA.$Zn^{2+}$/ATP** | **-175.87** | **-123.98** |
| **DPA.$Zn^{2+}$/ADP** | **-131.28** | **-75.09** |
| **DPA.$Zn^{2+}$/G6P** | **-178.20** | **-119.32** |
| **DPA.$Zn^{2+}$/$NaH_2PO_4$** | **-115.31** | **-65.01** |
| **DPA.$Zn^{2+}$/Adenosine** | **-103.48** | **-38.55** |

**F. DLS and zeta potential study**

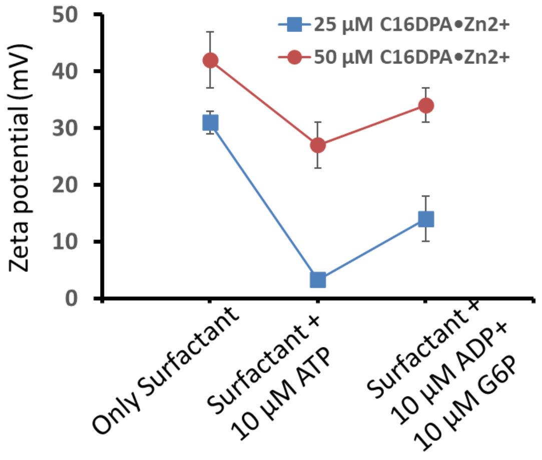


**Fig. S15.** Change in zeta potential profile of surfactant below cac concentration [25 µM] and above cac [50 µM] with ATP [10 µM] and ADP[10 µM] + G6P [10 µM]. Experimental conditions: 5mM HEPES buffer pH7, 25°C.

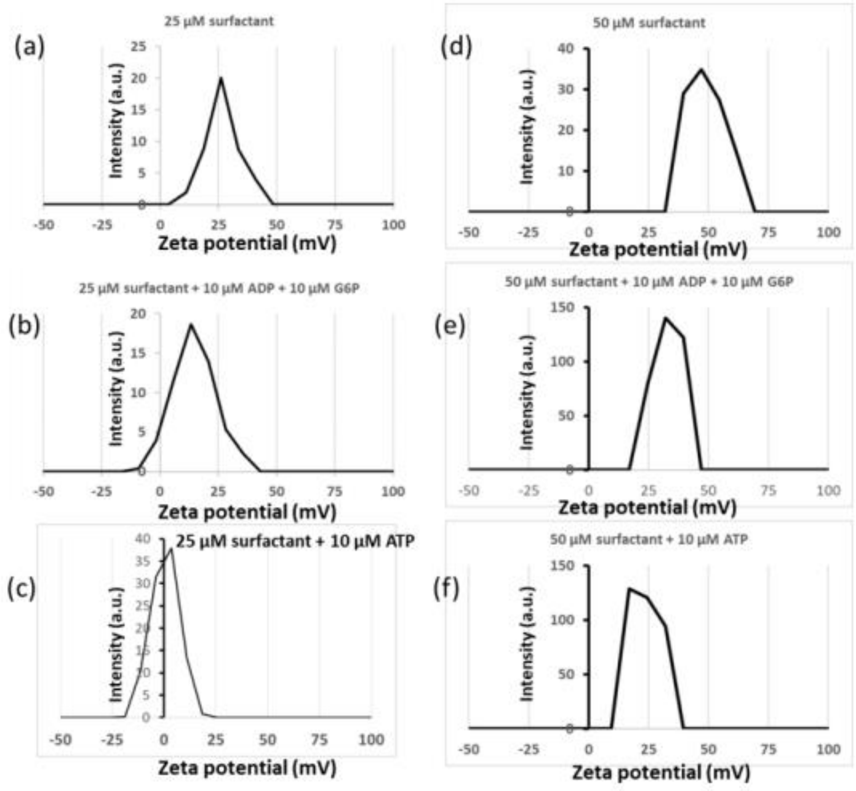


**Fig. S16.** Zeta potential profile of (a)25 µM surfactant (b) 25 µM surfactant+10 µM ADP+10 µM G6P (c) 25 µM surfactant+10 µM ATP (d) 50 µM $C_{16}$DPA.$Zn^{2+}$ (e) 50 µM $C_{16}$DPA.$Zn^{2+}$ +10 µM ADP+10 µM G6P (f) 50 µM $C_{16}$DPA.$Zn^{2+}$ +10 µM ATP. Experimental Conditions: [HEPES]=5mM, pH=7, T=25°C.

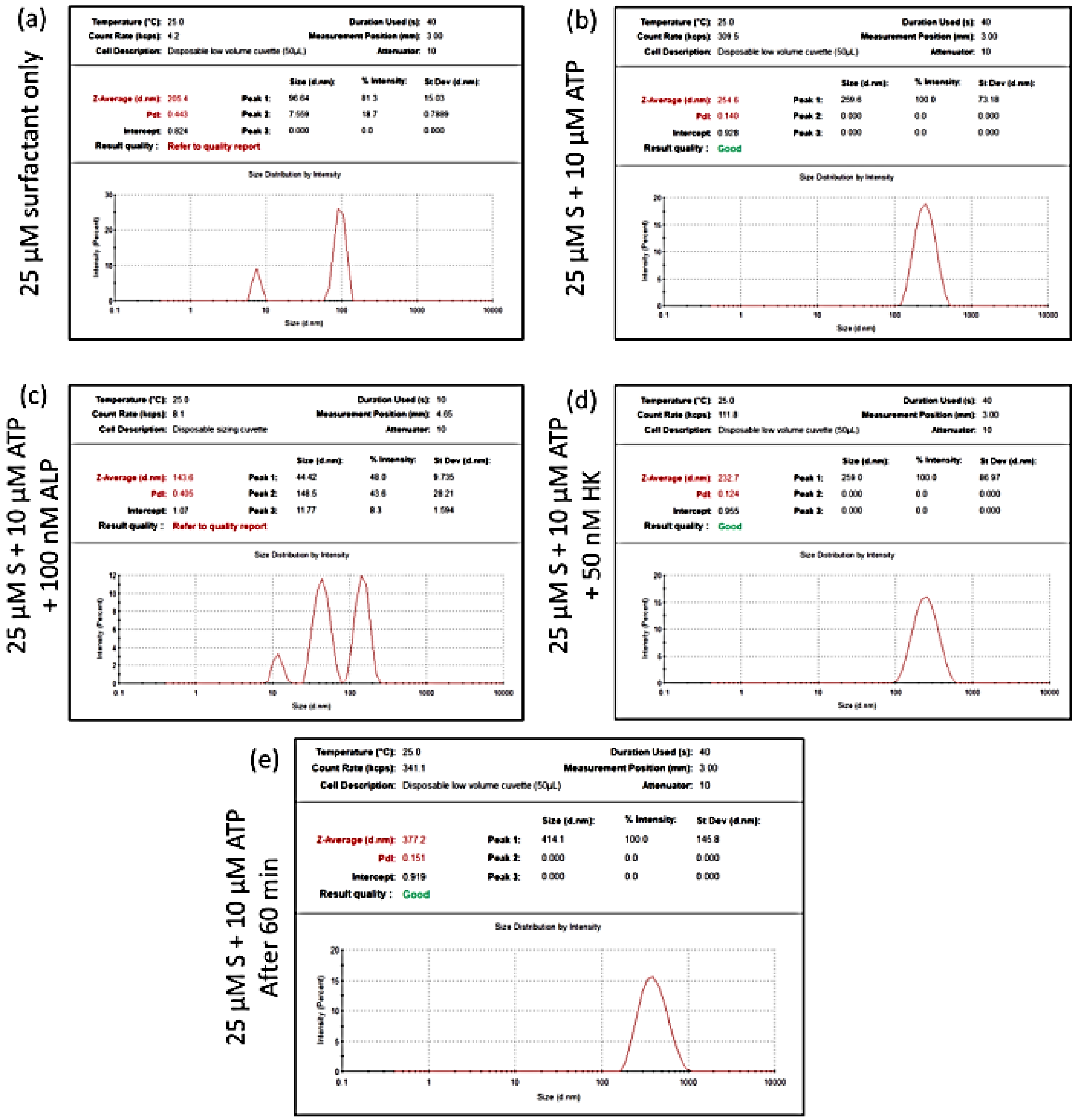


**Fig S17.** Hydrodynamic diameter of the assemblies measured with dynamic light scattering (DLS) (a) Surfactant (25 µM) only; (b) with 10 µM ATP and surfactant 25 µM, (c) 10 µM ATP at fixed surfactant conc. (25 µM) and in presence of 100nM ALP (d) Size of assemblies in presence of 25 µM surfactant with 10 µM ATP and 50nM HK, (e) with 10 µM ATP and surfactant 25 µM after 60 min of mixing. Experimental conditions: [Glucose] =1 mM, [$Mg(NO_3)_2$] = 500 µM, [HEPES] = 5 mM, pH = 7, T = 25°C. The measurements were taken after 60 minutes of reaction.

In Fig. S17, panel A represents data of only 25 µM surfactant where count rate is very low (4.2 kcps), high polydispersity index (PDI) and not reliable data for proper analysis. Panel B (25 µM surfactant + 10

μM ATP) and panel D (25 μM surfactant + 10 μM ATP + 50 nM HK) showed reliable data with low PDI (0.14 and 0.12) and high count rate of 309 and 111, respectively. Panel C (25 μM surfactant + 10 μM ATP + 50 nM ALP) showed unreliable data with high polydispersity (0.44 and 0.42) and low count rate of 8.1 kcps, similar to only surfactant, suggesting degradation of the ATP along with aggregate, whereas DLS data with HK showed stable structure.

**G. Transmission electronic microscopic (TEM) imaging**

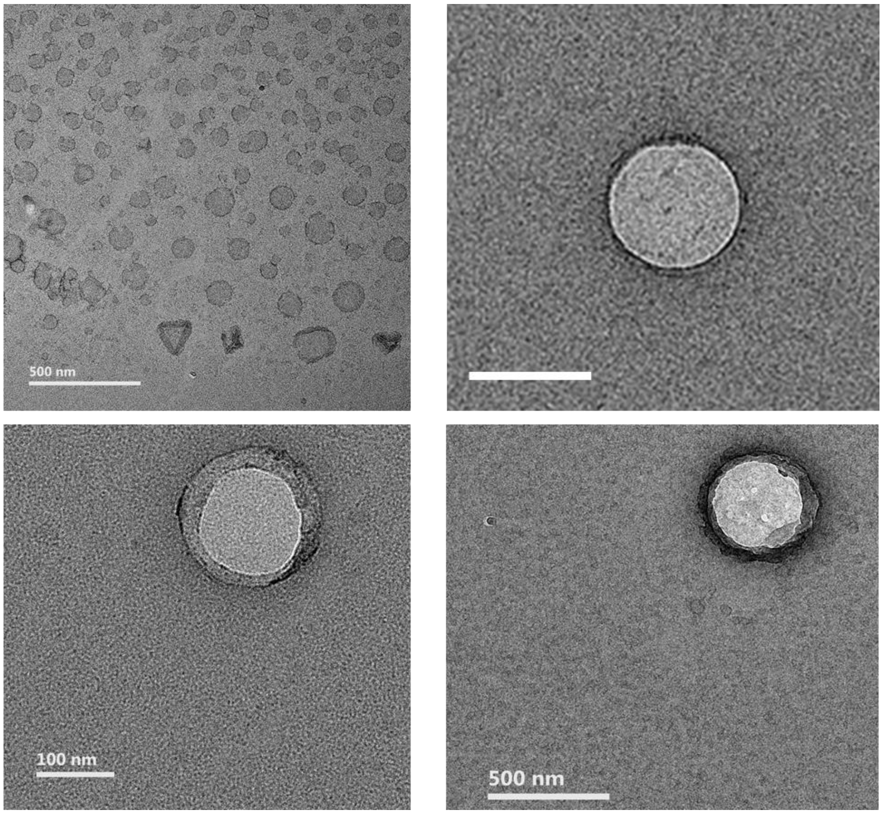


**Fig. S18.** TEM images of 50 μM surfactant + 10 μM ATP at different scales. Experimental Conditions: [HEPES]= 5mM, pH=7, T=25°C.

## H. Turbidity study

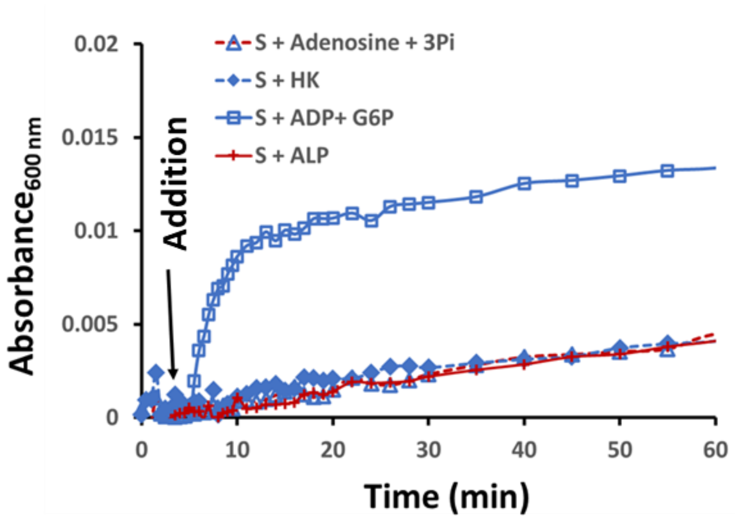


**Fig. S19.** Change in turbidity (at 600nm) as function of time upon addition of different enzyme and products to a solution of 25 µM surfactant, Glucose (1mM), $Mg(NO_3)_2$= 500 µM, the arrow denotes the addition of Adenosine + 3Pi/HK/ADP+G6P/ALP to the solution. Conditions: [HEPES]= 5mM, pH=7, T=25°C.

## I. Fluorescence Microscopic study

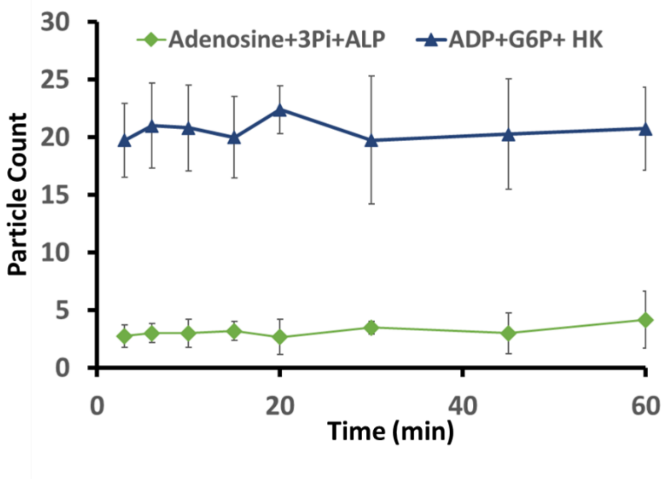


**Fig. S20.** Statistical analysis of the images of [$C_{16}DPA.Zn^{2+}$]=25 µM with Adenosine+3Pi+ALP, and ADP+G6P+HK. Experimental details: [C153] =2.5 µM, [Adenosine] = 10 µM, [Pi]= 30 µM, [ATP]= 10 µM. [Glucose]=1 mM, and [$Mg(NO_3)_2$]=500 µM [HEPES] = 5 mM, pH 7, T=25°C. Images were taken under

fluorescent microscope at 10 different positions in each zone at 40x zoom and the experiments were replicated 3 times to have an average of 30 images per zone.

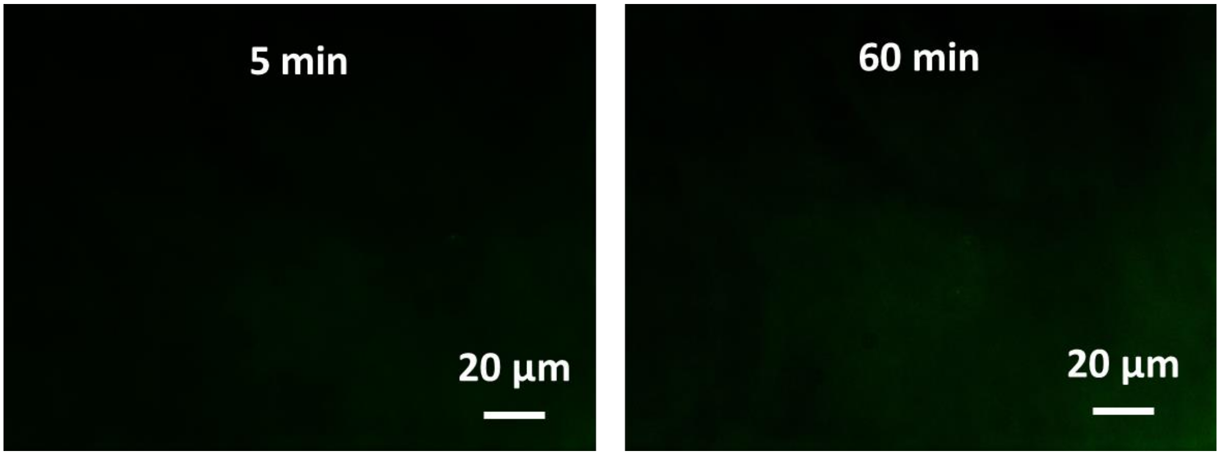


**Fig. S21.** Fluorescence microscopic images of Surfactant over time. Experimental details: [$C_{16}DPA{\cdot}Zn^{2+}$]= 25 µM, [C153]=2.5 µM, [Glucose]=1 mM, and [$Mg(NO_3)_2$]=500 µM [HEPES] = 5 mM, pH 7, T=25°C.

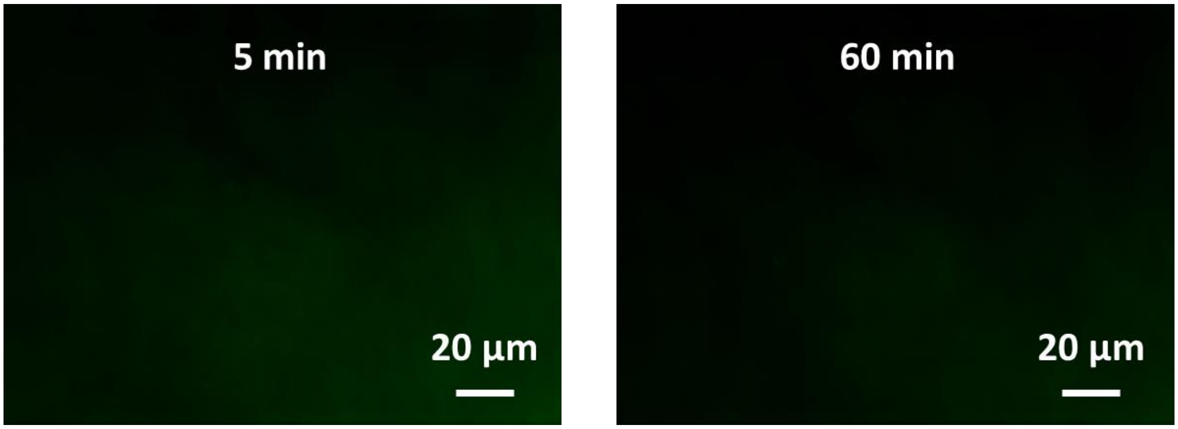


**Fig. S22.** Fluorescence microscopic images of Surfactant with ALP(100 nM) over time. Experimental details: $C_{16}DPA{\cdot}Zn^{2+}$ (25 µM), C153 (2.5 µM), Glucose=1mM, and $Mg(NO_3)_2$=500 µM [HEPES] = 5 mM, pH 7, T=25°C.

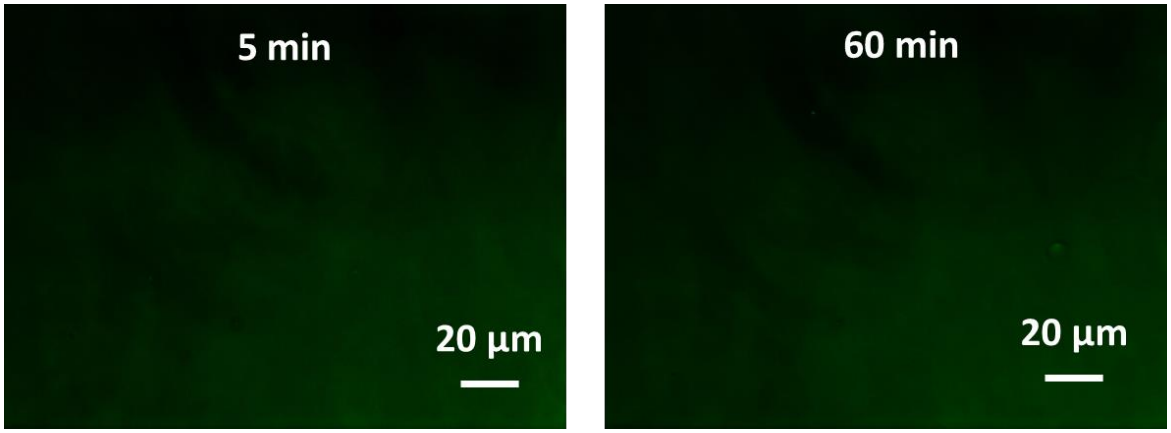


**Fig. S23.** Fluorescence microscopic images of Surfactant with HK(50 nM) over time. Experimental details: $C_{16}DPA{\cdot}Zn^{2+}$ (25 µM), C153 (2.5 µM), Glucose=1mM, and $Mg(NO_3)_2$=500 µM [HEPES] = 5 mM, pH 7, T=25°C.

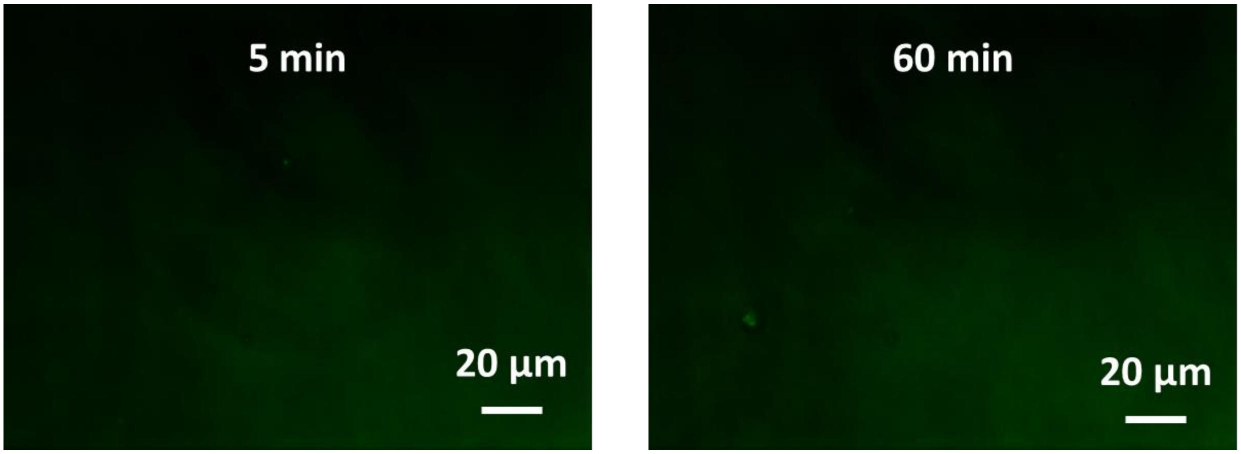


**Fig. S24.** Fluorescence microscopic images of Surfactant with Adenosine(10 µM) and 3Pi(30 µM) over time . Experimental details: [$C_{16}DPA{\cdot}Zn^{2+}$] =25 µM, [C153]=2.5 µM, [Glucose]=1 mM, and [$Mg(NO_3)_2$]=500 µM [HEPES] = 5 mM, pH 7, T = 25°C.

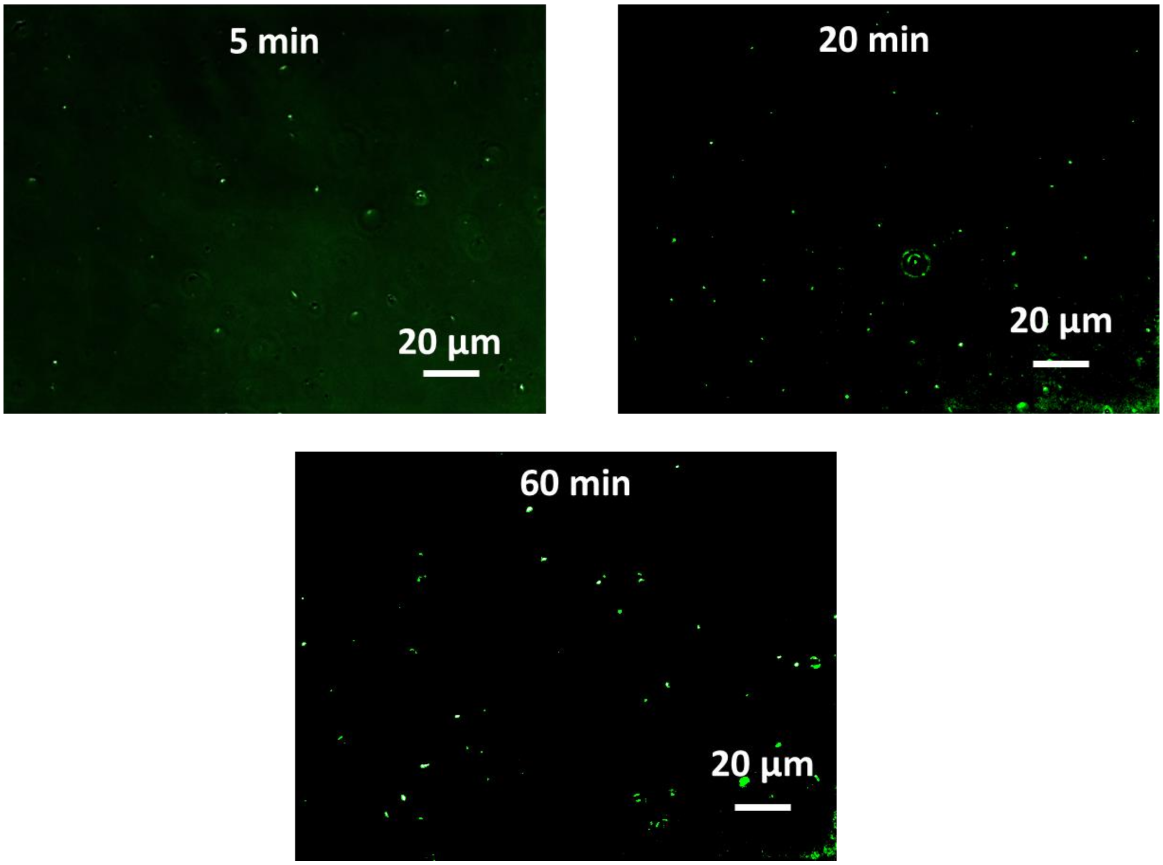


**Fig. S25.** Fluorescence microscopic images of Surfactant with ATP (10 µM) over time showing the appearance of fluorophore bound assemblies. Experimental details: [$C_{16}$DPA·$Zn^{2+}$] =25 µM, [C153] =2.5 µM, [Glucose] = 1 mM, and [$Mg(NO_3)_2$]=500 µM [HEPES] = 5 mM, pH 7, T=25°C.

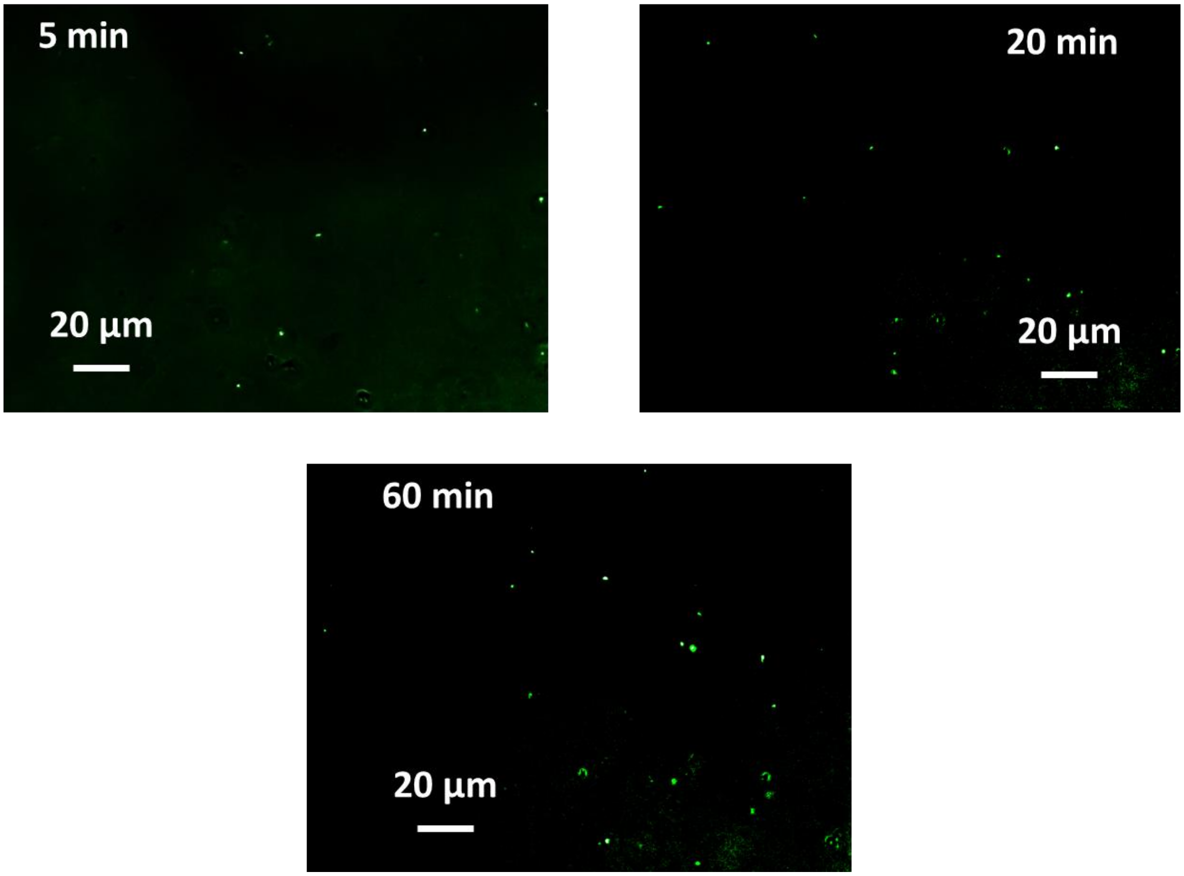


**Fig. S26.** Fluorescence microscopic images of Surfactant with ADP (10 µM), G6P (10 µM) and HK (50 nM), over time showing the appearance of fluorophore bound assemblies. Experimental details: [$C_{16}DPA{\cdot}Zn^{2+}$] =25 µM, [C153] = 2.5 µM, [Glucose] = 1 mM, and [$Mg(NO_3)_2$] = 500 µM [HEPES] = 5 mM, pH 7, T=25°C.

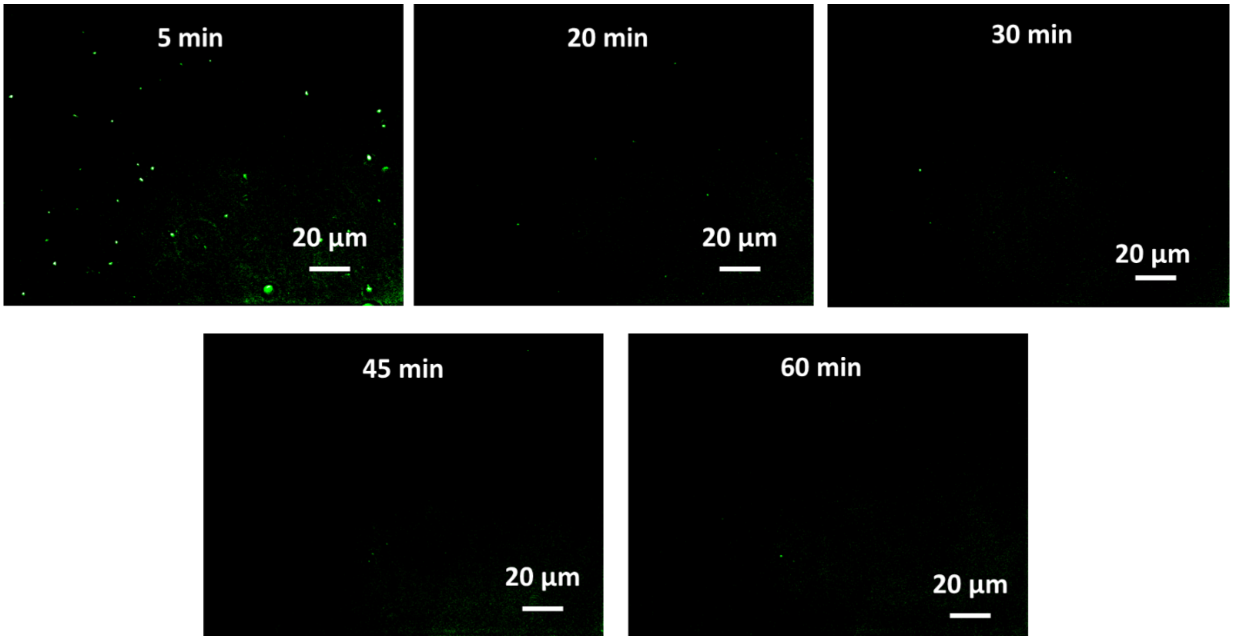


**Fig. S27.** Fluorescence microscopic images of Surfactant with ATP(10 µM) and ALP(100 nM), over time showing the appearance and disappearance of fluorophore bound assemblies. Experimental details: [$C_{16}$DPA·$Zn^{2+}$]=25 µM, [C153]=2.5 µM, [Glucose]=1 mM, and [$Mg(NO_3)_2$]=500 µM [HEPES] = 5 mM, pH 7, T=25°C.

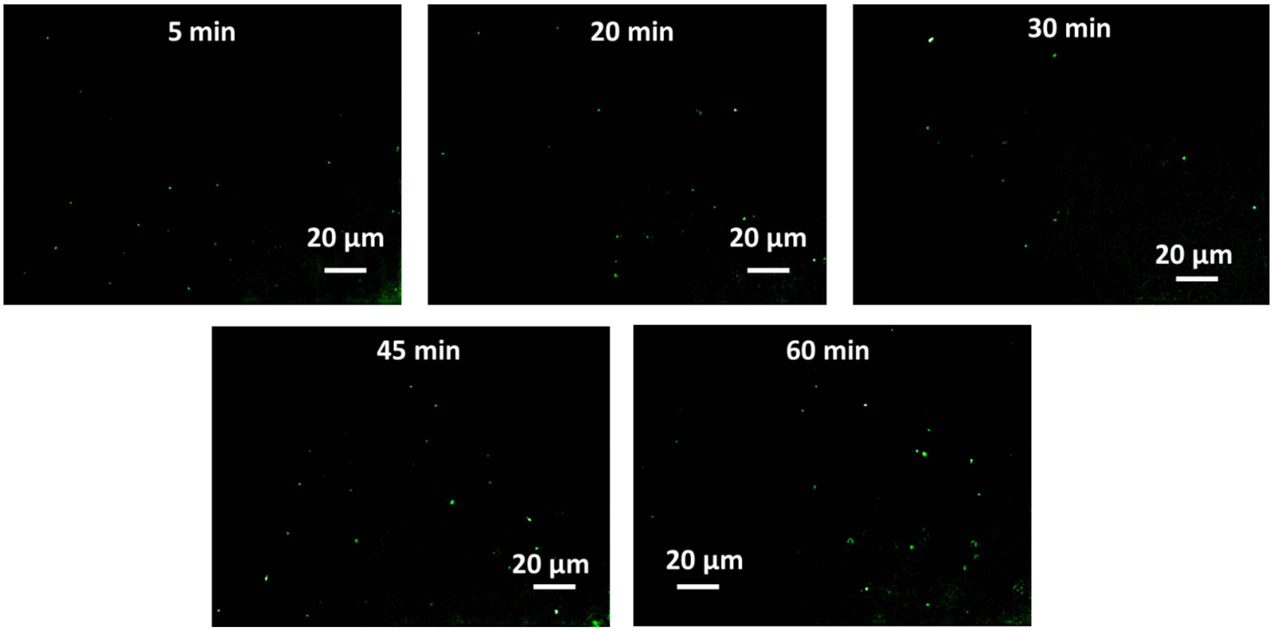


**Fig. S28.** Fluorescence microscopic images of Surfactant with ATP(10 µM) and HK (50 nM), over time showing the appearance of fluorophore bound assemblies. Experimental details: [$C_{16}$DPA·$Zn^{2+}$]= 25 µM, [C153] =2.5 µM, [Glucose]=1 mM, and [$Mg(NO_3)_2$]=500 µM [HEPES] = 5 mM, pH 7, T=25°C.

## J. Spatiotemporal study of assembly formation using fluid dynamics

To study the spatiotemporal evolution of $S_2T$ and $S_2P$ (mentioned in section B) under flow condition, a computational model was designed using MATLAB R2019b, FEATool Multiphysics. Here, we collectively studied the transport mechanism of both reactive and non-reactive species involved in system along with their rate of formation and deformation using computational fluid dynamics[S4-S6]. For this purpose, we designed our system so that T was distributed evenly on the squared grid while either S or S, and E were added from opposite boundaries as shown in figure S30 and figure 4a and 4d in main manuscript.

For describing the one-dimensional distribution of reactive species over space with time we have used Fick's second law of fluid dynamics so that

$$\frac{dC(x,t)}{dt} = D_x \frac{\partial^2 C}{\partial x^2} - V_x \frac{\partial C}{\partial x} \pm r_c \qquad \text{S10}$$

where C = the concentration of the species at position x and time t, $\boldsymbol{D_x}$ = diffusion coefficient, $\boldsymbol{V_x}$ = the linear flow velocity, and $\boldsymbol{r_c}$ = rate of change of species, while the '±' symbol denotes the rate of formation and deformation of species.

Following above equation, we rewrote the equations for each component of our system incorporating previously mentioned mass balance equations (S5-S9) in place of $\boldsymbol{r_c}$. Apart from this, Because of horizontal placement of grid, we considered the effect of diffusion on species movement and neglected the velocity term in equation S10.

Now, to solve our system of coupled differential equation under flow conditions, we first designed a square grid space containing 14,816 grid points and 294,912 triangular grid cells, an example is shown in figure S29.

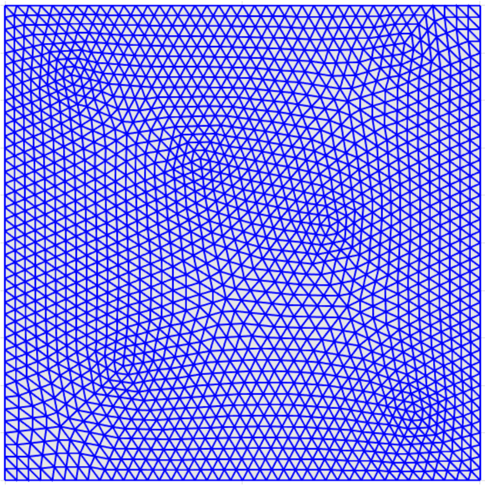

**Fig. S29.** A sample square grid space containing 1937 grid points and 3712 triangulations.

For initial conditions, we assumed that T (1 µM) is evenly distributed on grid. It is to be noted that here all the dimensions are unitless and we have assumed the unit of concentration and time as µM and hour, respectively for ease of understanding. Also, we assumed that diffusion rate of all assembled states is equal for simplification of equations. Firstly, we studied when monomer S was added from one side (boundary 4) of grid, while all other boundaries were left empty (fig. S30a). For solving the coupled equations, we marked the boundary conditions for all the species in accordance with Dirichlet and Neumann boundary conditions at all boundaries[S7]. For this case, the only equation to be followed was equation S1, as E was absent. Sample code containing used equations is shown in note S2.

For quantifying, $S_2T$ assembly formation by holding Neumann condition at boundary 4 and Dirichlet condition at boundary 2. The parameter fixed for $S_2T$ at boundary 4 was 1 µM/h, while 0.2 µM/h at boundary 2. Also, S followed Dirichlet condition as 1 µM/h, and 0.5 µM/h at boundary 4, and boundary 2, respectively. While solving this system of equation, over 5 h with 0.02-time steps following Crank-Nicolson time-step scheme, we observed that as time increases $S_2T$ formation increases. The maximum

$S_2T$ concentration can be seen at boundary 4 which gradually decreases as move towards boundary 2 (fig. 4b+c in main text, supplementary video SV1).

```
#NoteS2

#Matlab equations when S from boundary 4 and E from boundary 2 (derived from equations S5-S9)

u' - ux_x = m*m*w - u                    #S2T

w' - wx_x = - m*m*w + u                  #T

m' - mx_x  = - m*m*w + u                 #S
```

Note S2:

(a)

(b)

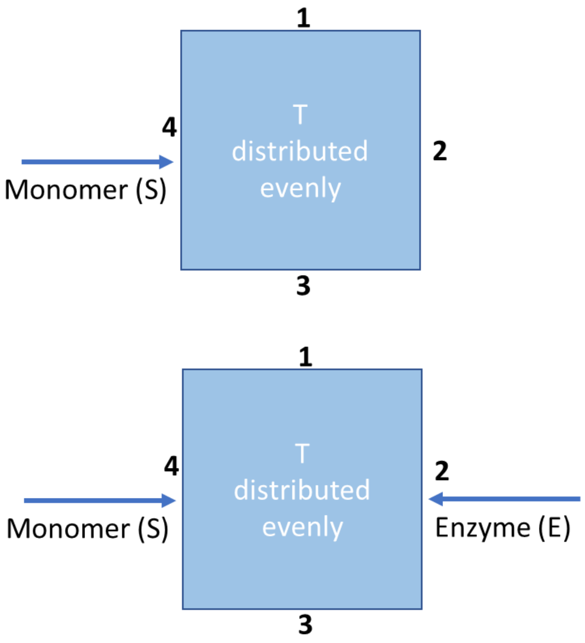


**Fig. S30.** Representative grid space where T is distributed evenly in space, and when (a) monomer, S was added from boundary 4; and (b) monomer S, was added from boundary 4, and enzyme, E was added from boundary 2 simultaneously.

```
#NoteS3

#Matlab equations when S from boundary 4 and E from boundary 1 (derived from equations S5-S9)


u' - ux_x = m*m*w - u - (u/1+u)                         #S2T

v' -  vx_x  = m*m*n - v                                 #S2P

w' - wx_x = - m*m*w + u - (w/1+w)                       #T

n' -  nx_x = (u/1+u) +  (w/1+w) - m*m*n + v             #P

m' - mx_x  = - m*m*w + u + (u/1+u) - m*m*n + v          #S
```

#Note S3

Apart from this, when we added S from boundary 4 and E from boundary 2, the set of equations holding were (S1) – (S4). The set of equations used in Matlab are shown in note S3. Here also, the parameters for S, R, and $S_2T$ were same and this set of equations was also solved in similar way as mentioned in previous paragraph. Additionally, the rate of product formation and product-driven dimeric assembly ($S_2P$) formation was added at respective boundaries.

For $S_2P$ formation over time, the Neumann boundary parameter was fixed at 0.5 µM/hour at boundary 2. While solving this set of equations over similar time constraint, we observed formation of $S_2T$ formation at boundary 4 which decreases gradually as we move towards boundary 4, while $S_2P$ assembly formation was more at boundary 2 which gradually decreases as we move towards boundary 4 (fig. 4d+e, supplementary video SV1, SV2).

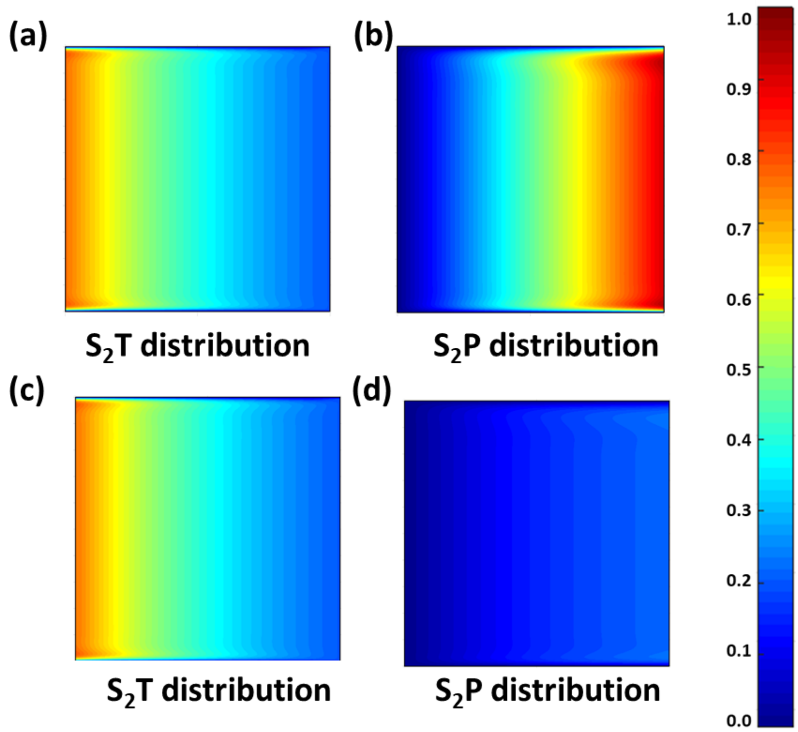


**Fig. S31.** Compositional distribution of dimeric assembly that is, (a)$S_2T$, and (c) $S_2P$, when overall rate of change of $S_2T$ formation on boundary 4 is 1 µM /h, and $S_2P$ formation on boundary 2 is 1 µM /h. Compositional distribution of (b) $S_2T$, and (c) $S_2P$, when overall rate of change of $S_2T$ formation on boundary 4 is 1 µM/h, and $S_2P$ formation on boundary 2 is 0.1 µM/h.

Additionally, we studied the effect of change of parameters on dimeric assembly formation. For this purpose, we varied the rate of change of $S_2P$ formation on boundary 2 to 1 µM/h, and 0.1 µM/h, while keeping rate of change of $S_2T$ formation constant at boundary 4 at 1 µM /h. (Fig. S31)

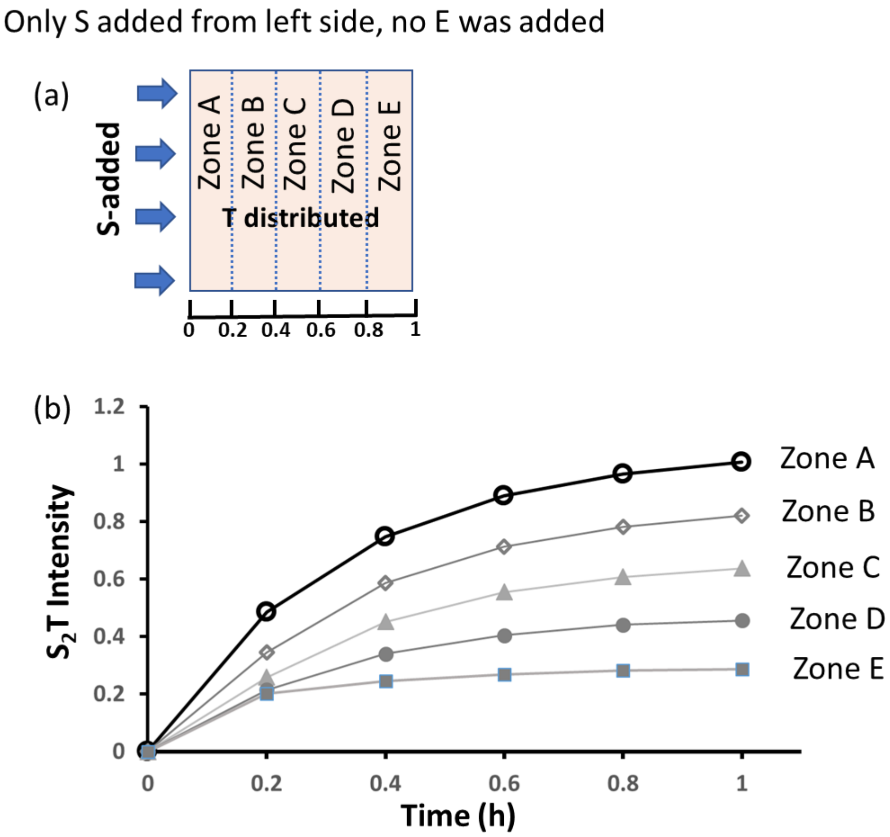


**Fig. S32.** (a) Grid space to show equivalent space zone A-E when T is distributed on space while S is added from left boundary from zone A. (b) Average intensity of zone A-E, at different time interval. Both intensity and time are dimensionless entity, here hour has been chosen as unit of time for logical comparison with experiment.

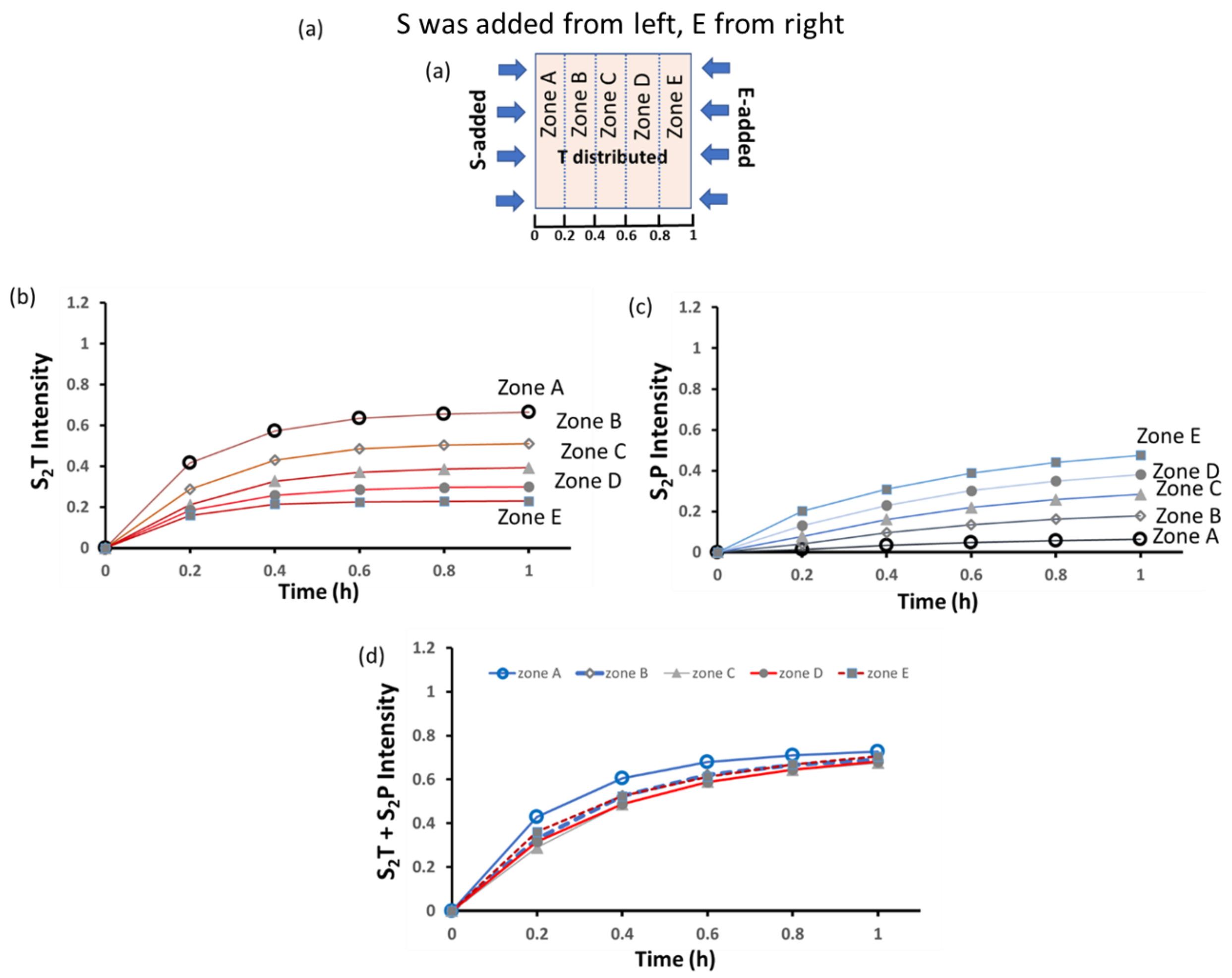


**Fig. S33.** (a) Square-shaped grid space to show equidistance space zone A-E when T is distributed on space while S is added from left boundary from zone A and E is added from right boundary from zone E. Average intensity of (b) $S_2T$, (c) $S_2P$ and (d) combined $S_2T$ and $S_2P$ in zone A-E, at different time interval. Both intensity and time are dimensionless entity, here hour has been chosen as unit of time for logical comparison with experiment.

**K. Experimental verification of spatiotemporal change in number of aggregates**

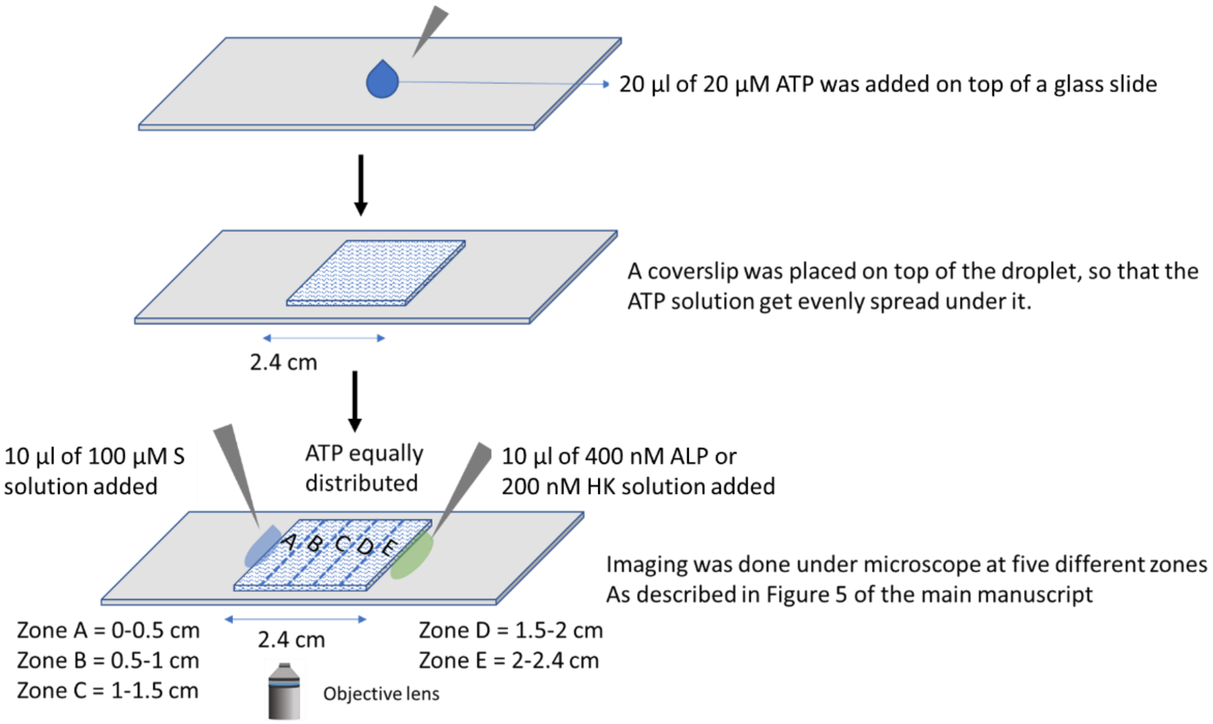


**Fig. S34.** Schematic representation of the experimental set up for the appearance and disappearance of assemblies over time in a space having concentration gradient of ATP and Enzyme.

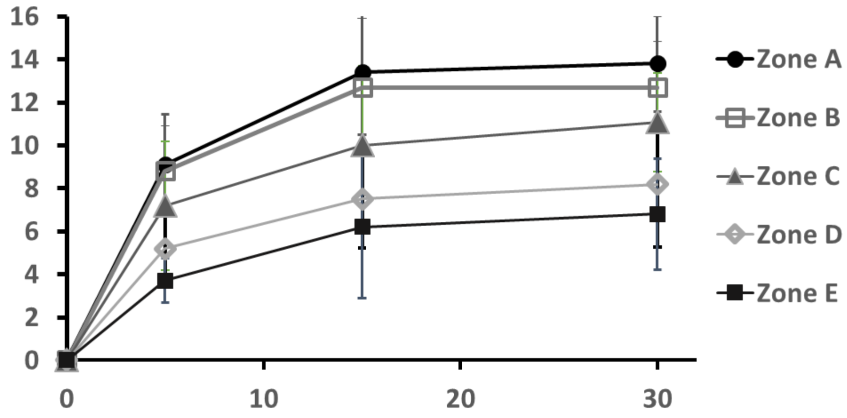


**Fig. S35.** Statistical analysis of the images taken at different positions after inserting the mixture of the $C_{16}DPA.Zn^{2+}$ (25 µM) with ATP (10 µM) at 5, 15 and 30 min. Experimental details: C153 (2.5 µM), Glucose=1mM, and $Mg(NO_3)_2$=500 µM [HEPES] = 5 mM, pH 7, T=25°C. Images were taken under fluorescent microscope at 10 different positions in each zone at 40x zoom and the experiments were replicated 3 times to have an average of 30 images per zone.

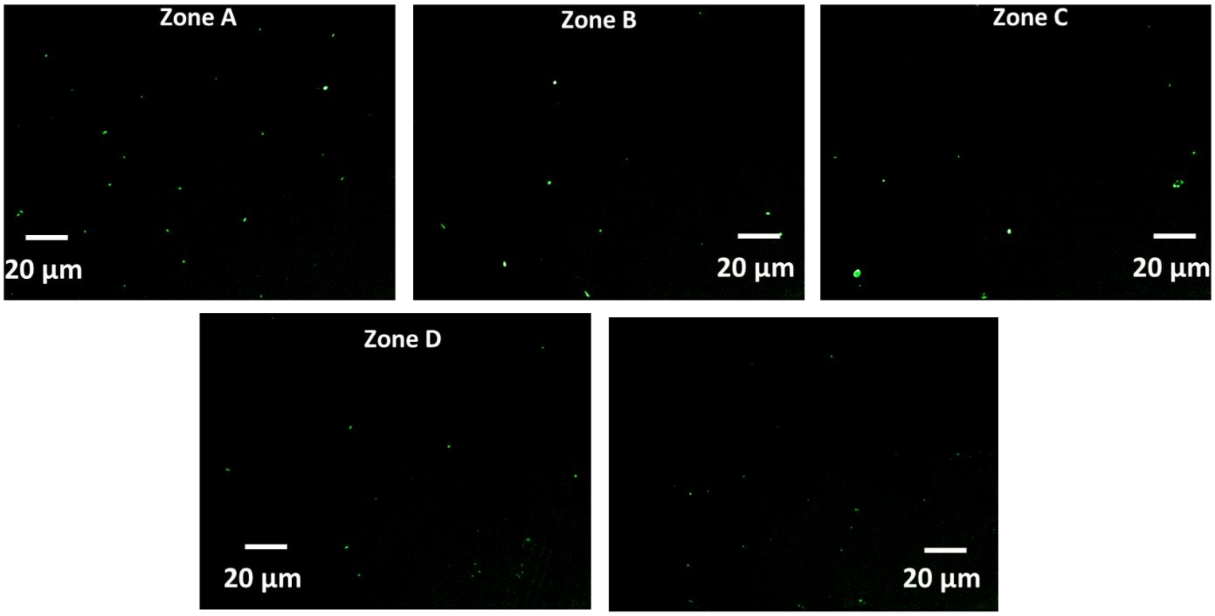


**Fig. S36.** Fluorescence microscopic images of assemblies formed over time in different spatial zones with concentration gradient of Surfactant while ATP was evenly distributed in space. Experimental details: [C153]= 2.5 μM, [$C_{16}$DPA.$Zn^{2+}$] = 25 μM, [ATP]= 10 μM, [Glucose]=1 mM, and [$Mg(NO_3)_2$]=500 μM [HEPES] = 5 mM, pH 7, T=25°C.

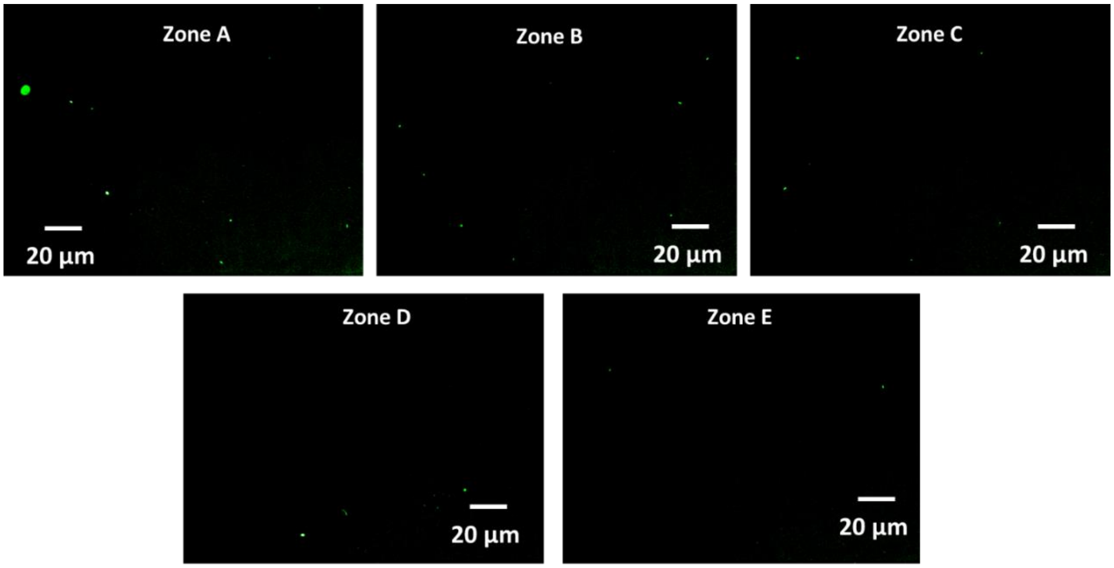


**Fig. S37.** Fluorescence microscopic images for the appearance and disappearance of assemblies over time in a space having concentration gradient of Surfactant and ALP where ATP is evenly distributed in space. Experimental details: [C153] =2.5 μM, [$C_{16}$DPA.$Zn^{2+}$] =25 μM, [ATP]= 10 μM, [ALP]= 100 nM, [Glucose]=1 mM, and [$Mg(NO_3)_2$]=500 μM [HEPES] = 5 mM, pH 7, T=25°C.

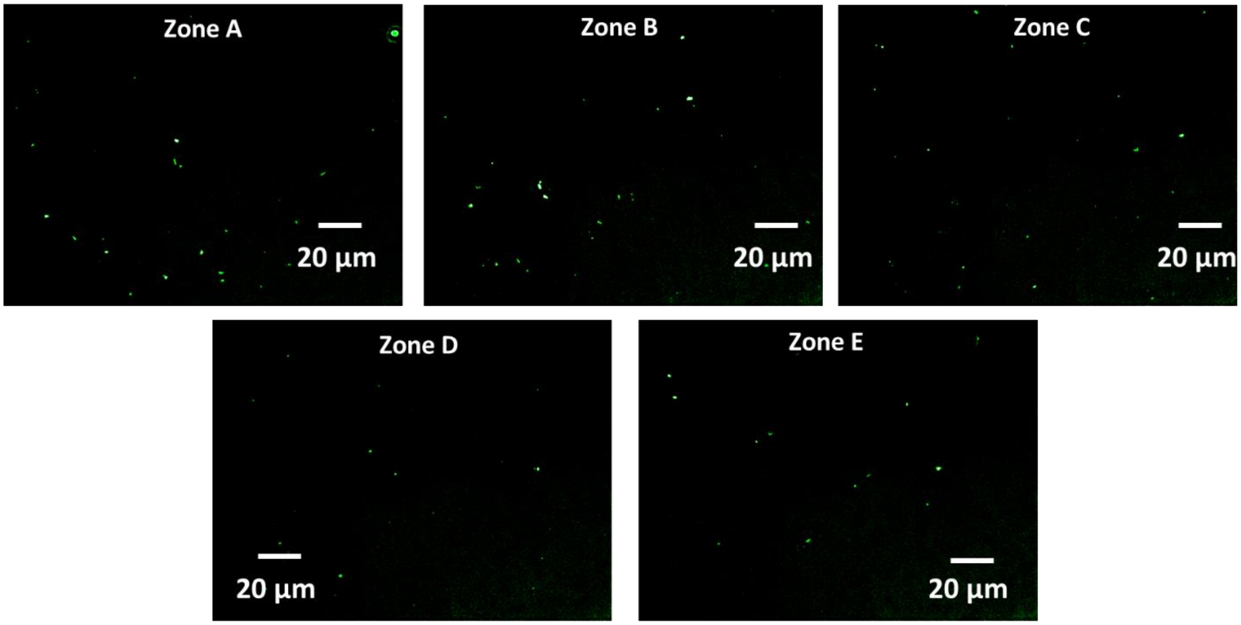


**Fig. S38.** Fluorescence microscopic images for the appearance of assemblies over time in a space having concentration gradient of Surfactant and Enzyme where ATP is evenly distributed in space. Experimental details: [C153] =2.5 µM, [$C_{16}$DPA.$Zn^{2+}$] =25 µM, [ATP]= 10 µM, [HK]= 50 nM, [Glucose]=1 mM, and [$Mg(NO_3)_2$]=500 µM [HEPES] = 5 mM, pH 7, T=25°C.

**Table S9.** Calculated P-values (in parenthesis) to determine the significance level of the counting of fluorescent particles between different zones for the image **Fig. S35** of the electronic supplementary information (ESI) for the data points taken at **5 min**. Here ATP was evenly distributed in all zones and only from zone A, surfactant was added. Here AB signifies comparison between zone A and B, similarly BC between zone B and C and so on.

| | | | |
|---|---|---|---|
| **AB** (0.81) | **AC** (0.14) | **AD** (0.0035) | **AE** (<0.0001) |
| | **BC** (0.15) | **BD** (0.0017) | **BE** (<0.0001) |
| | | **CD** (0.075) | **CE** (0.0006) |
| | | | **DE** (0.1) |

**Table S10.** Calculated P-values (in parenthesis) to determine the significance level of the counting of fluorescent particles between different zones for the image **Fig. S35** of the supplementary information for the data points taken at **30 min**. Here ATP was evenly distributed in all zones and only from zone A, surfactant was added. Here AB signifies comparison between zone A and B, similarly BC between zone B and C and so on.

| | | | |
|---|---|---|---|
| **AB** (0.18) | **AC** (0.0115) | **AD** (0.0005) | **AE** (0.0001) |
| | **BC** (0.055) | **BD** (0.0017) | **BE** (0.0002) |
| | | **CD** (0.16) | **CE** (0.096) |
| | | | **DE** (0.92) |

**Table S11.** Calculated P-values (in parenthesis) to determine the significance level of the counting of fluorescent particles between different zones for the image **Fig. 5b** of the main manuscript for the data points taken at **5 min**. Here ATP was evenly distributed in all zones and from zone A, surfactant and from zone E ALP enzyme was added. Here AB signifies comparison between zone A and B, similarly BC between zone B and C and so on.

| | | | |
|---|---|---|---|
| **AB** (0.5) | **AC** (0.07) | **AD** (0.0003) | **AE** (<0.0001) |
| | **BC** (0.26) | **BD** (0.0011) | **BE** (0.0001) |
| | | **CD** (0.006) | **CE** (0.0001) |
| | | | **DE** (0.14) |

**Table S12.** Calculated P-values (in parenthesis) to determine the significance level of the counting of fluorescent particles between different zones for the image **Fig. 5b** of the main manuscript for the data points taken at **30 min**. Here ATP was evenly distributed in all zones and from zone A, surfactant and from zone E ALP enzyme was added. Here AB signifies comparison between zone A and B, similarly BC between zone B and C and so on.

| | | | |
|---|---|---|---|
| **AB** **(0.58)** | **AC** **(<0.0001)** | **AD** **(<0.0001)** | **AE** **(<0.0001)** |
| | **BC** **(<0.0001)** | **BD** **(<0.0001)** | **BE** **(<0.0001)** |
| | | **CD** **(0.014)** | **CE** **(0.0026)** |
| | | | **DE** **(0.23)** |

**Table S13.** Calculated P-values (in parenthesis) to determine the significance level of the counting of fluorescent particles between different zones for the image **Fig. 5c** of the main manuscript for the data points taken at **5 min**. Here ATP was evenly distributed in all zones and from zone A, surfactant and from zone E, HK enzyme was added. Here AB signifies comparison between zone A and B, similarly BC between zone B and C and so on.

| | | | |
|---|---|---|---|
| **AB** **(0.85)** | **AC** **(0.1)** | **AD** **(0.0003)** | **AE** **(<0.0001)** |
| | **BC** **(0.14)** | **BD** **(0.0012)** | **BE** **(<0.0001)** |
| | | **CD** **(0.07)** | **CE** **(0.0062)** |
| | | | **DE** **(0.12)** |

**Table S14.** Calculated P-values (in parenthesis) to determine the significance level of the counting of fluorescent particles between different zones for the image **Fig. 5c** of the main manuscript for the data points taken at **30 min**. Here ATP was evenly distributed in all zones and from zone A, surfactant and from zone E, HK enzyme was added. Here AB signifies comparison between zone A and B, similarly BC between zone B and C and so on.

| | | | |
|---|---|---|---|
| **AB** (0.22) | **AC** (0.11) | **AD** (0.01) | **AE** (0.006) |
| | **BC** (0.64) | **BD** (0.09) | **BE** (0.06) |
| | | **CD** (0.19) | **CE** (0.15) |
| | | | **DE** (0.96) |

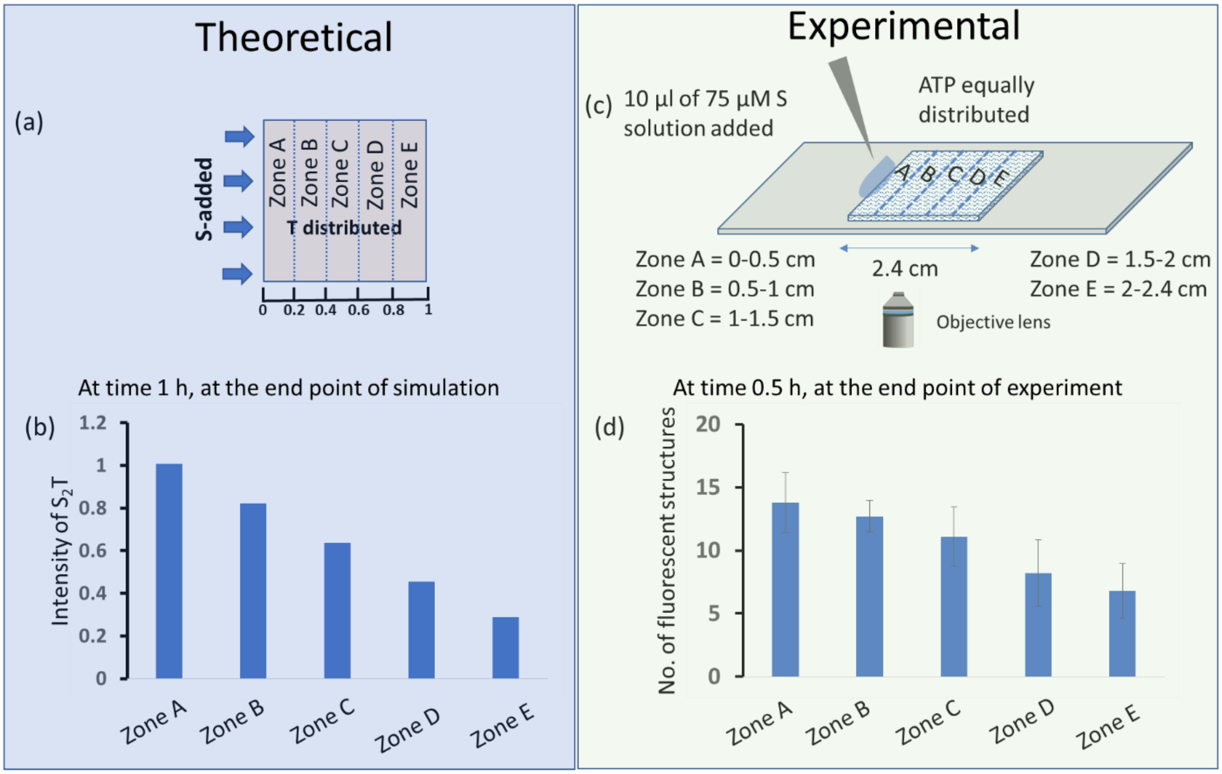


**Fig. S39.** (a and c) A comparative study between theory and experiment when only S was added from the left side when T is distributed through the space (for theoretical modelling) and surfactant was added from the left side when ATP was distributed under the cover slip (for experiment). (b and d) Both assembled structure $S_2T$ (from theory) and number of self-assembled unit for surfactant and ATP assembly (from experiment) were plotted across zones A to E at the end point of simulation or experiment.

From this data, decreasing trend of structures from zone A to E has been observed in both cases.

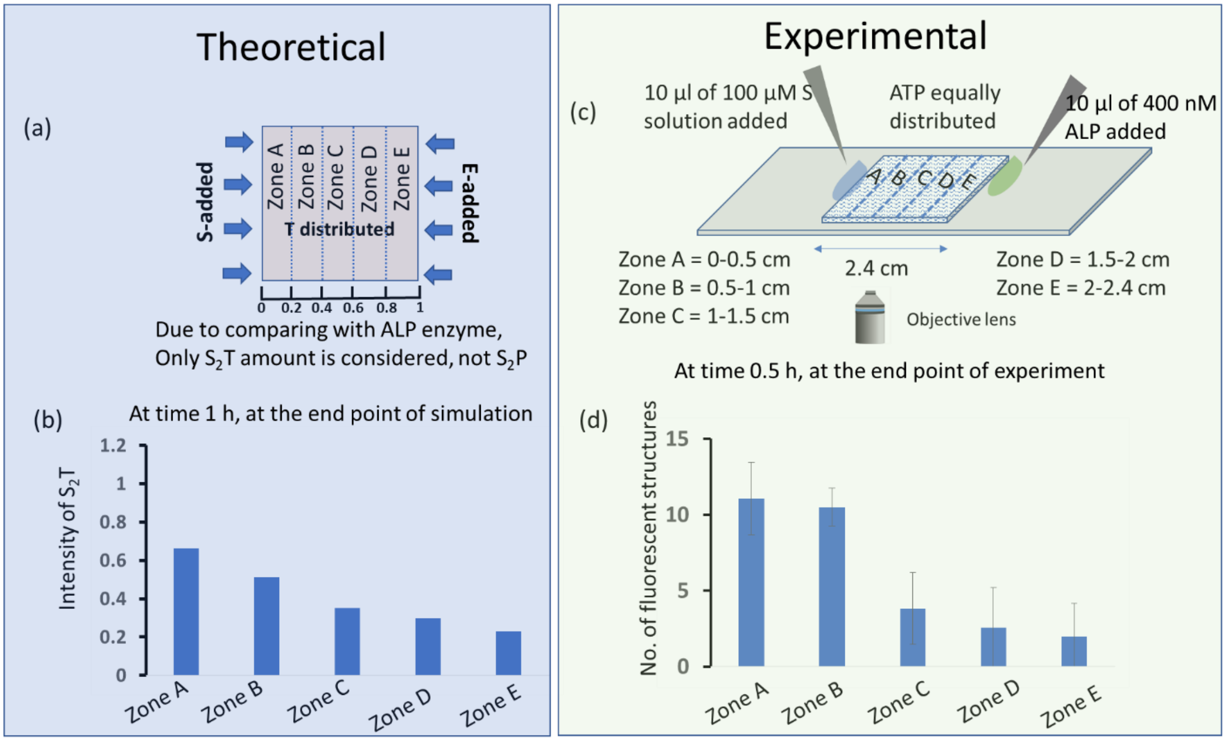


**Fig. S40.** (a and c) A comparative study between theory and experiment when S was added from the left side and E from the right when T is distributed through the space (for theoretical modelling) and surfactant was added from the left side and ALP was added from right when ATP was distributed under the cover slip (for experiment). (b and d) Both assembled structure $S_2T$ (from theory) and number of self-assembled unit for surfactant and ATP assembly (from experiment) were plotted across zones A to E at the end point of simulation or experiment.

From this data, decreasing trend of structures and also much lower number structure in all zones compared to without E have been observed (see Fig. S39). The population decreased from zone A to E has been observed in both cases, where in experiment zone A and B showed higher number of structures, compared to zone C to E. Similar kind of trend was also observed in theoretical trend.

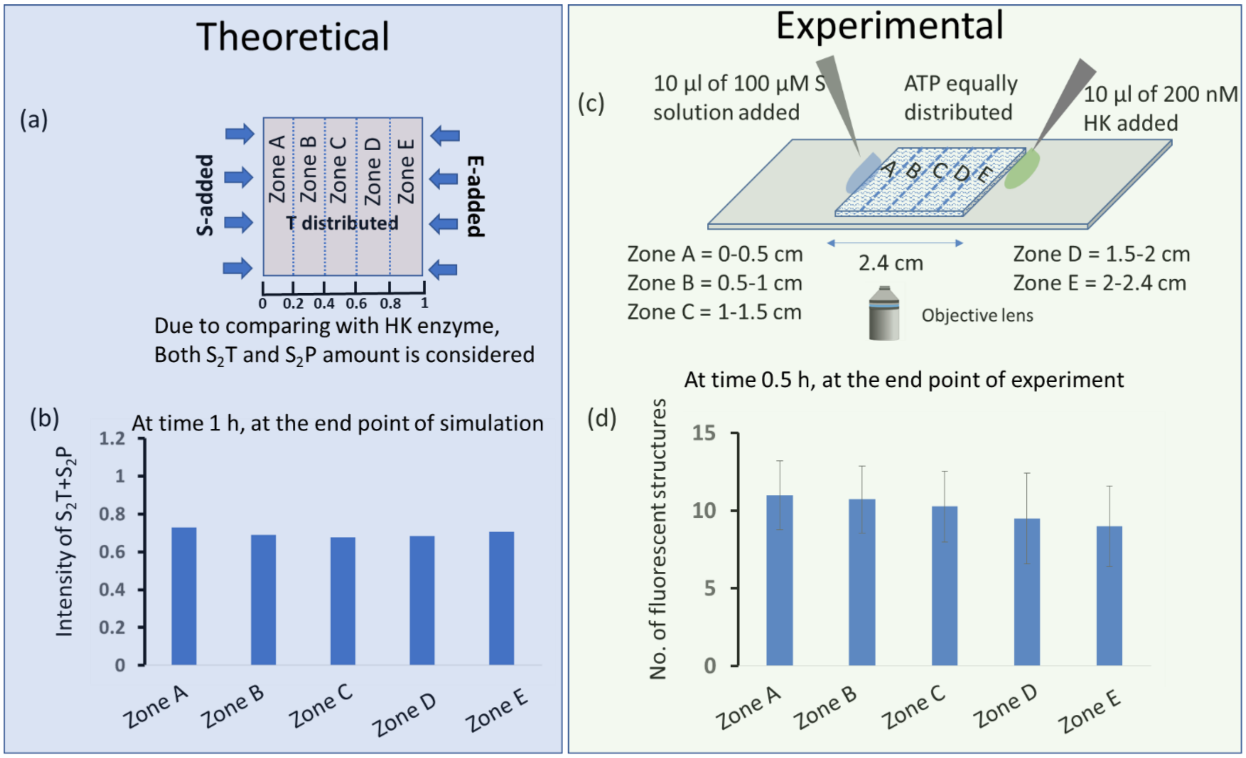


**Fig. S41.** (a and c) A comparative study between theory and experiment when S was added from the left side and E from the right when T is distributed through the space (for theoretical modelling) and surfactant was added from the left side and HK was added from right when ATP was distributed under the cover slip (for experiment). (b and d) Both assembled structure $S_2T$ (from theory) and number of self-assembled unit for surfactant and ATP assembly (from experiment) were plotted across zones A to E at the end point of simulation or experiment.

From this data, almost equal number of structures in all zones have been observed, both in experiment and theory.

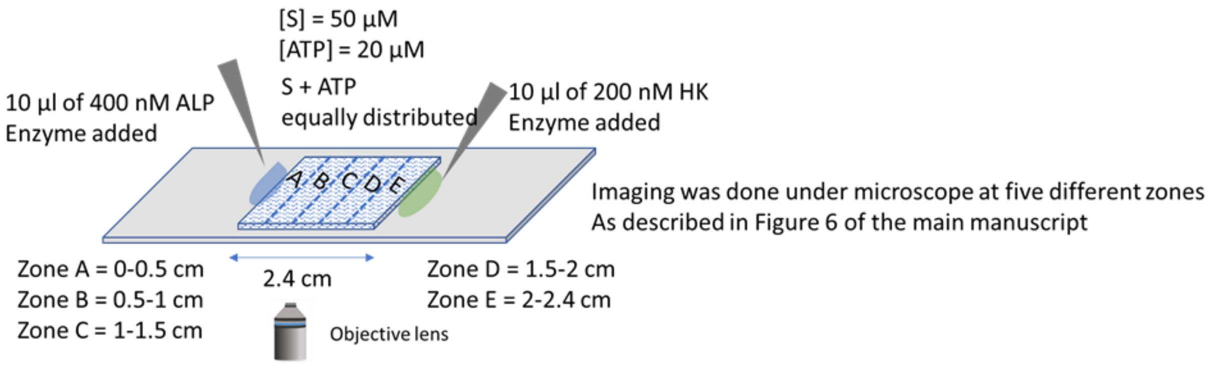


**Fig. S42.** Schematic representation of the experimental set up when Surfactant + ATP assemblies are distributed equally under the coverslip mixed with glucose and $Mg(NO_3)_2$ and ALP and HK were added from two opposite sides of the droplet.

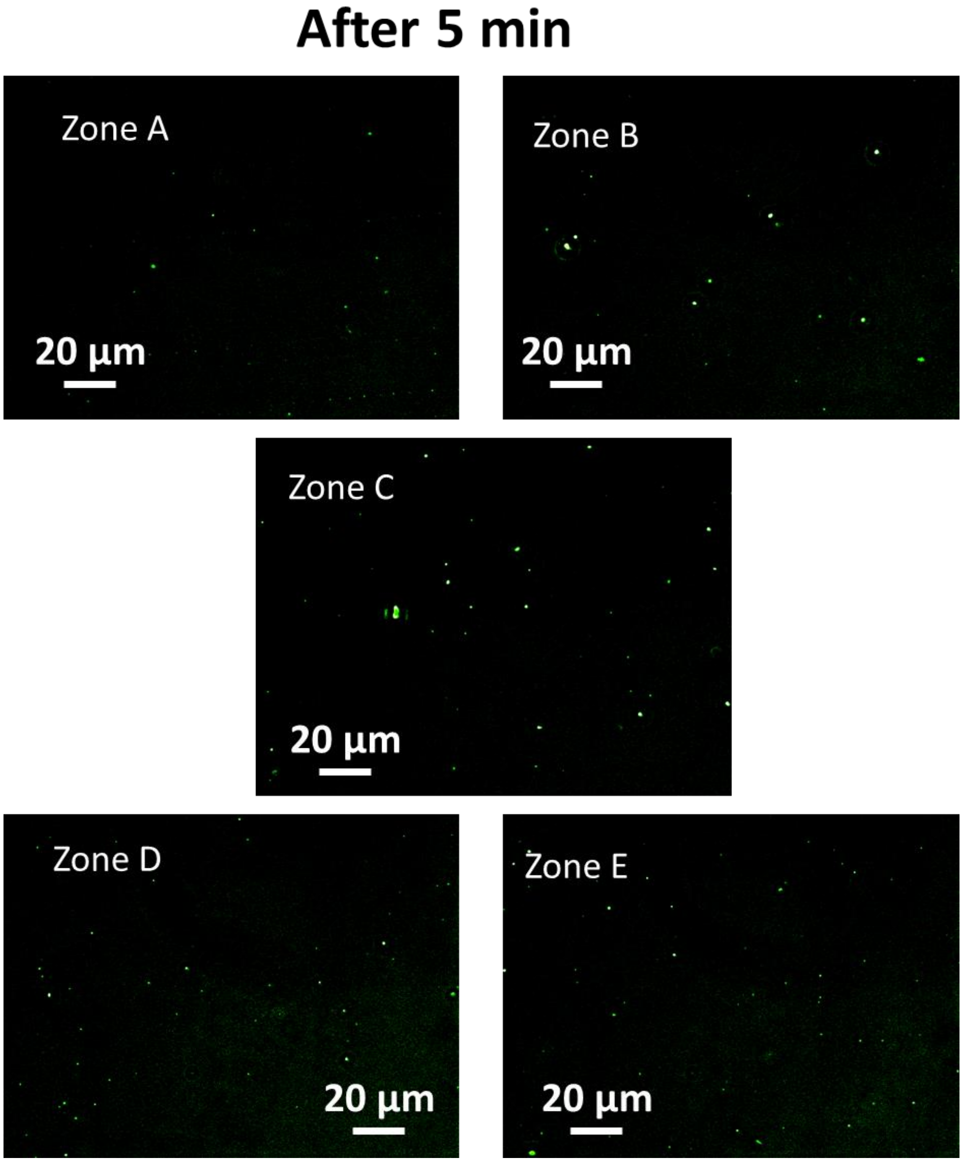


**Fig. S43.** Fluorescence microscopic images for the appearance and disappearance of assemblies after 5 min time interval in a space having concentration gradient of Enzyme ALP and HK from zone A and E, respectively when surfactant + ATP aggregates were evenly distributed in space. Experimental details: [C153] =2.5 µM, [$C_{16}$DPA.$Zn^{2+}$] = 50 µM, [ATP] = 20 µM, [ALP] = 400 nM, [HK]= 200 nM, [Glucose]=1 mM, and [$Mg(NO_3)_2$]=500 µM, [HEPES] = 5 mM, pH 7, T=25°C.

# After 30 min

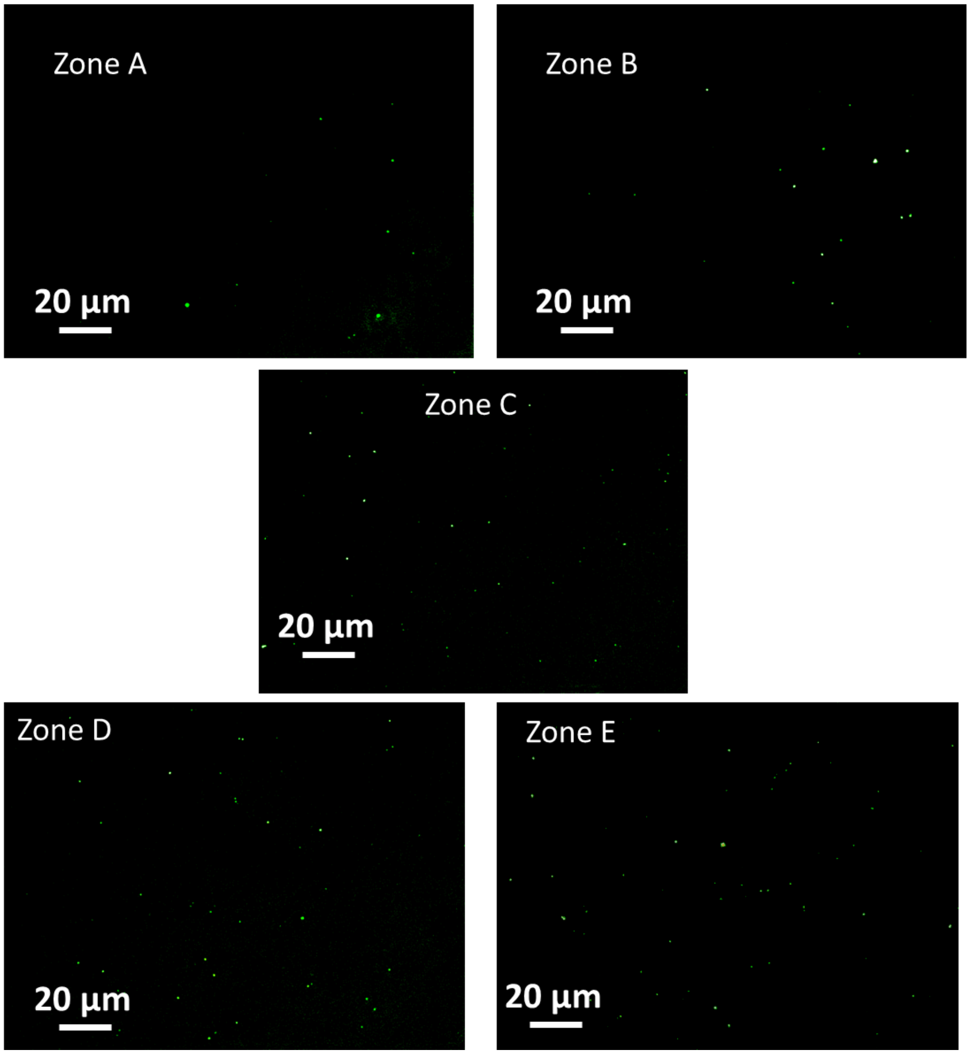


**Fig. S44.** Fluorescence microscopic images for the appearance and disappearance of assemblies after 30 min time interval in a space having concentration gradient of Enzyme ALP and HK from zone A and E, respectively when surfactant + ATP aggregates were evenly distributed in space. Experimental details: [C153] =2.5 µM, [$C_{16}DPA.Zn^{2+}$] = 50 µM, [ATP] = 20 µM, [ALP] = 400 nM, [HK]= 200 nM, [Glucose]=1 mM, and [$Mg(NO_3)_2$]=500 µM, [HEPES] = 5 mM, pH 7, T=25°C.

**L. Supporting video**

SV1. Spatiotemporal formation of S2T over grid when monomer, S was added from boundary 4 (left side).

SV2. Spatiotemporal formation of S2T over grid when monomer, S was added from boundary 4 (left side), and enzyme, E was added from boundary 2 (right side).

SV3. Spatiotemporal formation of $S_2P$ over grid when monomer, S was added from boundary 4 (left side), and enzyme, E was added from boundary 2 (right side).

**N. References**

S1. E. Shandilya and S. Maiti, *ChemSystemsChem,* 2020, **2**, e190040.

S2. R. Feng, Y. Xu, H. Zhao, X. Duan and S. Sun, *Analyst*, 2016, **141**, 3219-3223.

S3. M. J. Frisch, G. W. T., H. B. Schlegel, G. E. Scuseria,M. A. Robb, J. R. Cheeseman, G. Scalmani, V. Barone, B. Mennucci, G. A. Petersson, H. Nakatsuji, M. Caricato, X. Li, H. P. Hratchian,; A. F. Izmaylov, J. B., G. Zheng, J. L. Sonnenberg, M. Hada,; M. Ehara, K. T., R. Fukuda, J. Hasegawa, M. Ishida, T. Nakajima,; Y. Honda, O. K., H. Nakai, T. Vreven, J. A. Montgomery, Jr.,; J. E. Peralta, F. O., M. Bearpark, J. J. Heyd, E. Brothers,; K. N. Kudin, V. N. S., T. Keith, R. Kobayashi, J. Normand,; K. Raghavachari, A. R., J. C. Burant, S. S. Iyengar, J. Tomasi,; M. Cossi, N. R., J. M. Millam, M. Klene, J. E. Knox, J. B. Cross,; V. Bakken, C. A., J. Jaramillo, R. Gomperts, R. E. Stratmann,; O. Yazyev, A. J. A., R. Cammi, C. Pomelli, J. W. Ochterski,; R. L. Martin, K. M., V. G. Zakrzewski, G. A. Voth,; P. Salvador, J. J. D., S. Dapprich, A. D. Daniels,; O. Farkas, J. B. F., J. V. Ortiz, J. Cioslowski, D. J. Fox, Gaussian, Inc., Wallingford CT. 2010.

S4. D. S. Raje and V. Kapoor, *Environ. Sci. Technol*. 2000, **34**, 1234-1239.

S5. M. E. Potter, L.-M. Armstrong and R. Raja, *Catal. Sci. Technol*. 2018, **8**, 6163-6172.

S6. C. M. Gramling, C. F. Harvey and L. C. Meigs*, Environ. Sci. Technol*. 2002, **36**, 2508-2514.

S7. E. Bormashenko, M. Frenkel, A. Vilk, I. Legchenkova, A. A. Fedorets, N. E. Aktaev, L. A. Dombrovsky and M. Nosonovsky, *Entropy* 2018, **20**, 956.
